# Lysine-Specific Demethylase 1 Inhibitors: A Comprehensive Review Utilizing Computer-Aided Drug Design Technologies

**DOI:** 10.3390/molecules29020550

**Published:** 2024-01-22

**Authors:** Di Han, Jiarui Lu, Baoyi Fan, Wenfeng Lu, Yiwei Xue, Meiting Wang, Taigang Liu, Shaoli Cui, Qinghe Gao, Yingchao Duan, Yongtao Xu

**Affiliations:** 1 School of Medical Engineering, Xinxiang Medical University, Xinxiang 453003, China; hd@xxmu.edu.cn (D.H.); lujiarui@xxmu.edu.cn (J.L.);; 2 Henan International Joint Laboratory of Neural Information Analysis and Drug Intelligent Design, Xinxiang 453003, China; 3 Xinxiang Key Laboratory of Biomedical Information Research, Xinxiang 453003, China; 4 School of Forensic, Xinxiang Medical University, Xinxiang 453003, China; 5 School of Pharmacy, Xinxiang Medical University, Xinxiang 453003, China

**Keywords:** LSD1/KDM1A inhibitor, computer-aided drug design, molecular docking, QSAR, molecular dynamics simulation

## Abstract

Lysine-specific demethylase 1 (LSD1/KDM1A) has emerged as a promising therapeutic target for treating various cancers (such as breast cancer, liver cancer, etc.) and other diseases (blood diseases, cardiovascular diseases, etc.), owing to its observed overexpression, thereby presenting significant opportunities in drug development. Since its discovery in 2004, extensive research has been conducted on LSD1 inhibitors, with notable contributions from computational approaches. This review systematically summarizes LSD1 inhibitors investigated through computer-aided drug design (CADD) technologies since 2010, showcasing a diverse range of chemical scaffolds, including phenelzine derivatives, tranylcypromine (abbreviated as TCP or 2-PCPA) derivatives, nitrogen-containing heterocyclic (pyridine, pyrimidine, azole, thieno[3,2-b]pyrrole, indole, quinoline and benzoxazole) derivatives, natural products (including sanguinarine, phenolic compounds and resveratrol derivatives, flavonoids and other natural products) and others (including thiourea compounds, Fenoldopam and Raloxifene, (4-cyanophenyl)glycine derivatives, propargylamine and benzohydrazide derivatives and inhibitors discovered through AI techniques). Computational techniques, such as virtual screening, molecular docking and 3D-QSAR models, have played a pivotal role in elucidating the interactions between these inhibitors and LSD1. Moreover, the integration of cutting-edge technologies such as artificial intelligence holds promise in facilitating the discovery of novel LSD1 inhibitors. The comprehensive insights presented in this review aim to provide valuable information for advancing further research on LSD1 inhibitors.

## 1. Introduction

Histone is a basic protein found in chromosomes that can bind to DNA. The protein can be subject to various types of modifications such as methylation, acetylation and phosphorylation, which control the expression of genes [1,2,3]. Previously, the methylation modification of histone was thought to be irreversible until 2004, when Professor Yang Shi discovered the first histone lysine-specific demethylase 1 (LSD1) [4].

**Figure 1:**
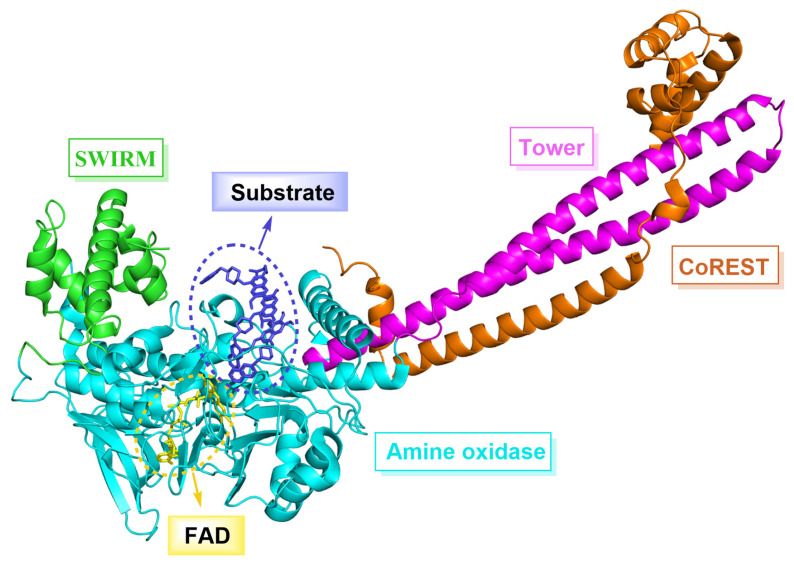
The structure of LSD1 (PDB code: 6TUY) composed of three major domains, namely the N-terminal SWIRM domain (green), Tower domain (pink) and the C-terminal amino oxidase-like (AOL) domain (cyan).

LSD1, also known as KDM1A, belongs to the monoamine oxidase (MAO) family and shares a 17.6% sequence similarity with monoamine oxidases A and B (MAO-A, MAO-B), as well as a 22.4% similarity with polyamine oxidase (PAO) [5,6]. LSD1 is composed of 852 amino acid residues and forms three major domains (Figure 1), namely the N-terminal SWIRM domain, the Tower domain and the C-terminal amino oxidase-like (AOL) domain. Specifically, the SWIRM domain contains six α helices that interact with chromatin [7]. The Tower domain has two inverted α helices that can bind to CoREST protein to maintain the overall structural stability of LSD1 [8]. The AOL domain is subdivided into a flavin adenine dinucleotide (FAD) binding domain and a substrate binding domain, both of which constitute the catalytic active center. During the catalytic process, FAD is initially oxidized [9], and then LSD1 is able to selectively remove monomethyl and dimethyl at H3K4 and H3K9 sites under the action of FAD, which plays a crucial role in regulating histone modifications [10,11,12] and gene expression mediated by hormone receptors [13,14]. In addition, LSD1 can also demethylate a variety of non-histone substrates such as p53 [15], DNA methyltransferase 1 (DNMT1) [16], signal transducer and activator of transcription 3 (STAT3) [17], E2F1 [18] and myosin phosphatase target subunit 1 (MYPT1) [19]. Multiple studies have shown that the overexpression of LSD1 is associated with various cancers (prostate cancer [20], breast cancer [21], liver cancer [22], stomach cancer [23], acute myeloid leukemia [24], etc.) and non-cancer diseases (blood diseases [25], cardiovascular diseases [26], etc.). In tumor cells, the overexpression of LSD1 induces abnormal cell proliferation, thereby facilitating the rapid growth and extensive dissemination of tumors. Effectively inhibiting the expression or function of LSD1 can mitigate the proliferation and dissemination of tumor cells, thereby markedly enhancing the therapeutic efficacy against cancer. Consequently, LSD1 has emerged as a compelling target for the development of anti-tumor drugs [27,28]. Despite LSD1 inhibitors’ extensive research, few have reached the market.

Traditional research and the development of new drugs is a long-cycle, high-risk and large investment process. On average, the development of a new drug takes about 10–15 years, and the investment cost is about USD 800 million [29,30,31]. However, the emergence of computer-aided drug design (CADD) technology has changed this landscape. The application of CADD technology can guide the rational development of new drugs, reduce blindness and contingency, speed up the drug development process and, ultimately, save human, material and financial resources. With the development of computing power and efficient algorithms, CADD techniques have been widely and successfully applied in the field of drug discovery, rapidly advancing the development of related drugs [32,33,34]. For example, in the research of protease inhibitors for the treatment of hepatitis C, a combination of computational and experimental methods has been used to design and synthesize a number of inhibitors with good inhibitory activity, including boceprevir, which has been approved as a direct-acting antiviral drug for the treatment of HCV [35]. As powerful auxiliary means for the rapid development of new drugs, some CADD techniques, including structure-based drug design (such as molecular docking and molecular dynamics simulations) and ligand-based drug design methods (such as 3D-QSAR and pharmacophore model screening), together with some artificial intelligence (AI) technologies, have also been applied in the research of LSD1 inhibitors.

In this review, we systematically summarize the research works on LSD1 inhibitors involving computational simulation techniques since 2010. To facilitate subsequent investigations of various inhibitors, this article organizes its review based on the logical progression of inhibitor types and their research developments. The relevant contents could provide valuable information for further research on LSD1 inhibitors. According to the molecular structural characteristics, these LSD1 inhibitors can be classified as phenelzine derivatives, tranylcypromine (abbreviated as TCP or 2-PCPA) derivatives, nitrogen-containing heterocyclic (pyridine, pyrimidine, azole, thieno[3,2-b]pyrrole, indole, quinoline and benzoxazole) derivatives, natural products (including sanguinarine, phenolic compounds and resveratrol derivatives, flavonoids and other natural products) and others (including thiourea compounds, Fenoldopam and Raloxifene, (4-cyanophenyl)glycine derivatives, propargylamine and benzohydrazide derivatives and inhibitors discovered through AI techniques).

## 2. Phenelzine Derivatives

**Figure 2:**
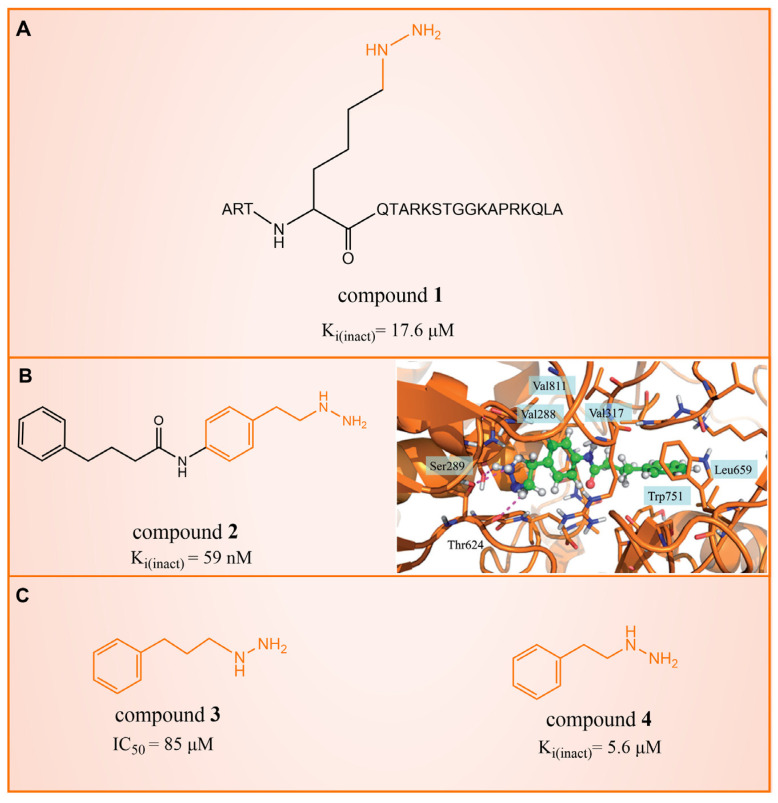
The structures of phenelzine derivatives. (**A**) Compound **1**; K_i(inact)_ is the apparent maximum inactivation rate. (**B**) Compound **2** and the binding mode with LSD1; the key amino acids are illustrated, and orange dash lines represent the hydrogen bond interactions (Reprinted with permission from Ref. [40]. Copyright 2015 Taylor & Francis). (**C**) Compound **3** and compound **4**; IC_50_ is half maximal inhibitory concentration.

In 2006, Lee et al. initially reported the modest inhibitory effects of hydrazine-containing MAO inhibitors and phenelzine on LSD1 [36]. However, a significant breakthrough occurred in 2010, when Cole et al. [37] identified a highly potent hydrazine-containing substrate analog, LSD1 irreversible inhibitor compound **1** (depicted in Figure 2A, with an apparent maximum inactivation rate (K_i(inact)_) of 17.6 μM; the name of the compound in the original paper is compound 18, and the names in brackets below are the names of the corresponding compounds in the original literatures) using a sensitive Amplex Red coupled assay [38]. In 2014, Cole’s group extended their research by designing a series of phenelzine derivatives based on the previous works [39]. Subsequent to this, Zhang et al. conducted molecular docking and molecular dynamics simulation studies on a series of phenelzine derivatives developed by Cole’s group in 2015 [40]. The molecular docking technology is a computational chemical method that is used to study the interactions between molecules and predict how they combine. The working principle of this technique is to simulate the binding of ligands (usually potential drug molecules) with proteins to determine their possible binding modes and minimum energy states [41]. This is helpful to reveal the interactions between ligands and proteins, and provides important information for drug design and optimization. Molecular docking technology plays a key role in drug discovery, which is helpful to speed up the process of discovering and developing new drugs [42]. Molecular dynamics (MD) simulation can predict the movement of each atom in protein or other molecular systems with time, based on the physical model that controls the interaction between atoms, and can simulate the movement and interaction of molecules in organisms to further verify the efficacy and safety of drugs [43,44,45]. This technology provides information about the molecular structure and conformation of drugs, which is helpful in optimizing drug design, improving drug efficacy and reducing side effects [46,47]. The molecular simulation methods above were employed to investigate the binding mode of phenethylhydrazine derivatives to LSD1. Through the molecular docking result of compound **2** (Figure 2B, K_i(inact)_ = 59 nM, compound **12d**) with LSD1 (Figure 2B) using Autodock Vina (https://vina.scripps.edu, accessed on 4 February 2015), they found that the hydrazine moiety of the inhibitor was able to form one hydrogen bond with Thr624 and two hydrogen bonds with the hydroxyl of Ser289. These interactions were deemed crucial for the effective binding of the inhibitor to the protein. Moreover, the benzene ring and alkyl chain portions of compound **2**, in addition to being able to form hydrophobic interactions with several hydrophobic residues including Val288, Val317, Val811 and Ala814, were able to establish extensive interactions with the surrounding hydrophobic residues, Leu659, Trp751 and Tyr761, reinforcing the overall binding. Utilizing the MM/GBSA (Molecular Mechanics Generalized Born Surface Area) method, based on molecular mechanics simulations, the binding free energy of protein–ligand complexes was predicted. The results, as presented in Table 1, revealed that compared to compound **3** (Figure 2C, ${\mathrm{IC}}_{50}^{\mathrm{LSD}1}$ = 85 μM, ΔG = −27.35 ± 0.32 kcal/mol, compound **9a**) and compound **4** (Figure 2C, K_i(inact)_ = 5.6 μM, ΔG = −34.13 ± 0.48 kcal/mol, phenelzine), compound **2** exhibited a lower binding free energy (ΔG = −50.04 ± 0.43 kcal/mol). On the cellular experimental level, compound **2** increased the signal of cell H3K4Me2, the EC_50_ value was as low as 2 μM, and it had a considerable dose–response effect on lung cancer cell line H460. Lung cancer cell line A549 and breast cancer cell line MDA-MB-231 also showed that H3K4Me2 increased its response to strangeness. This suggests that future investigations into phenelzine-based LSD1 inhibitors should retain the fundamental scaffold, especially the phenelzine moiety, and preserve the spatial orientation of key functional groups to ensure the effective binding of designed inhibitors to LSD1. Phenelzine derivatives are a useful probe for the continuous evaluation of LSD1 under physiological and pathological conditions. Through molecular docking and molecular dynamics simulation technology, it is helpful to analyze the binding mode and affinity between LSD1 and phenelzine inhibitors, and reveal the key interaction between LSD1 and phenelzine inhibitors, which provides new ideas for the development of LSD1 inhibitors in the future. 

## 3. Tranylcypromine (TCP/2-PCPA) Derivatives

**Figure 3:**
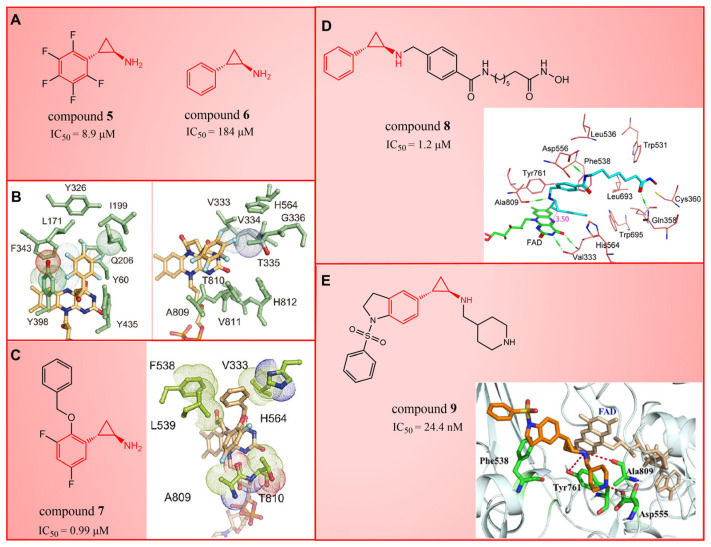
The structures of tranylcypromine derivatives. (**A**) Compounds **5** and **6**; (**B**) the co-crystal structural comparison of MAO-B/compound **5** complex and LSD1/compound **5** complex (reprinted with permission from Ref. [50] Copyright 2010 American Chemical Society); (**C**) Compound **7** and the 2-PCPA benzene ring formed stable hydrophobic interactions with the surrounding residues (reprinted with permission from Ref. [50] Copyright 2010 American Chemical Society); (**D**) compound **8** and predicted binding model of compound **8** with LSD1 (reprinted with permission from Ref. [51]. Copyright 2017 Elsevier); (**E**) compound **9** and complex structure of LSD1 upon binding to compound **9** (reprinted with permission from Ref. [52]. Copyright 2022 American Chemical Society).

In 2010, Mimasu et al. made a noteworthy observation regarding compound **5** (Figure 3A, ${\mathrm{IC}}_{50}^{\mathrm{LSD}1}$ = 8.9 μM, 2-PFPA). Through the superimposition of the co-crystal structures of LSD1/compound **5** and MAO-B/compound **5** (PDB IDs: 2Z5U [48] and 1OJB [49], respectively), they found that compound **5** formed a five-membered ring at C(4)a and N(5) of FAD in LSD1, whereas it only fused at the C(4)a position in MAO-B [50]. This structural insight prompted the design of 2-PCPA derivatives with functional groups strategically placed on the benzene ring of compound **6** (Figure 3A, ${\mathrm{IC}}_{50}^{\mathrm{LSD}1}$ = 184 μM, 2-PCPA) to enhance selectivity for LSD1. Subsequent activity screening led to the identification of compound **7** (Figure 3C, ${\mathrm{IC}}_{50}^{\mathrm{LSD}1}$ = 0.99 μM, S2101), which exhibited a smaller structure with potent inhibitory effects on LSD1. The binding mode analysis revealed that the ortho-located benzene ring and meta-positioned fluorine atom of the 2-PCPA benzene ring formed stable hydrophobic interactions with surrounding residues, including Val333, Phe538, Leu539, His564, Ala809 and Thr810. These interactions bolstered the binding stability of the complex. In 2017, our group found that inhibiting LSD1 and HDACs simultaneously exhibited anti-tumor effects. To develop dual inhibitors of LSD1/HDACs, a series of TCP derivatives were synthesized [51]. One of these compounds, compound **8** (Figure 3D, compound **7**), emerged as a promising candidate, displaying good inhibitory activity against LSD1 (${\mathrm{IC}}_{50}^{\mathrm{LSD}1}$ = 1.2 μM), HDAC1 (${\mathrm{IC}}_{50}^{\mathrm{HDAC}1}$ = 15 nM) and HDAC2 (${\mathrm{IC}}_{50}^{\mathrm{HDAC}2}$ = 23 nM). A molecular docking analysis (software: MOE 2015.10, Figure 3D) illustrated that the hydroxamic acid carbonyl group in compound **8** could establish hydrogen bonds with Gln358, and the amine group of its amide could form a hydrogen bond with Asp556. Furthermore, the amine group of TCP could form a salt bridge with Ala809, while its cyclopropylamine part could form hydrophobic interactions with the flavin ring, His564, Val333, Thr335, Thr310, Phe538 and Trp695. In brief, the position of compound **8** within LSD1 was well-suited to the activity pocket, providing valuable insights for designing novel dual inhibitors of LSD1/HDACs. In 2022, Li et al. undertook a comprehensive analysis of the structure–activity relationship of indole-5-yl-cyclopropane amine derivatives [52]. The introduction of a piperidine group was found to enhance LSD1 inhibitory activity. Notably, compound **9** (Figure 3E, ${\mathrm{IC}}_{50}^{\mathrm{LSD}1}$ = 24.4 nM, compound **7e**) exhibited robust inhibitory effects on LSD1. The dilution analysis suggested that compound **9** might interact covalently with the LSD1 enzyme. Molecular docking demonstrated that compound **9** could bind to the active region of LSD1 (Figure 3E), with its indole group forming a π–π stacking interaction with Phe538. Additionally, the protonated amine of the piperidinyl group formed a salt bridge with the negatively charged side chain Asp555, and also formed a hydrogen bond with Ala809 and Tyr761, respectively. Compound **9** showed strong anti-proliferative activities on MV-4-11 acute myeloid leukemia (AML) cell lines, induced the differentiation of AML cell lines and up-regulated the expression level of differentiation marker gene CD86 (EC_50_ = 470 nM). As of now, seven TCP-like inhibitors (Tranylcypromine, IMG-7289, ORY-1001, ORY-2001, GSK-2879552, INCB059872 and TAK-418; among them, many clinical trials of GSK-2879552, INCB059872 and TAK-418 were terminated due to safety and off-target effects) have entered clinical trials, underscoring the clinical potential of irreversible LSD1 inhibitors. However, overcoming the challenges associated with the irreversible properties of such inhibitors, leading to side effects and selectivity issues, remains an ongoing clinical hurdle.

## 4. Nitrogen-Containing Heterocyclic Derivatives

### 4.1. Pyridine Derivatives

**Figure 4:**
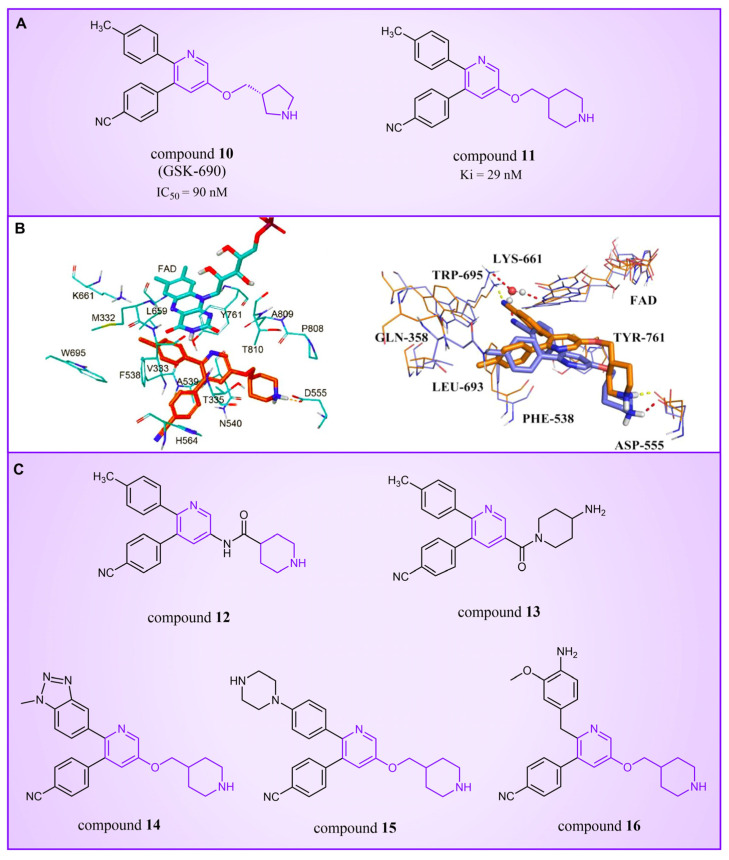
The structures of pyridine derivatives. (**A**) Compound **10** (GSK-690) and compound **11**; (**B**) complex structure of LSD1 upon binding to compound **11** ((**left**), PDB code: 2V1D, reprinted with permission from Ref. [55]. Copyright 2016 American Chemical Society) and superposition of molecular docking result with the average structure during MD of compound **11** ((**right**), reprinted with permission from Ref. [56]. Copyright 2018 Taylor & Francis); (**C**) compounds **12**–**16**.

Compound **10** (Figure 4A, GSK-690), a pyridine-based LSD1 reversible inhibitor, was unveiled by researchers from GlaxoSmithKline during the 2013 American Association for Cancer Research (ACCR) meeting [53]. Hitchin et al. demonstrated its effectiveness in inhibiting LSD1 (${\mathrm{IC}}_{50}^{\mathrm{LSD}1}$ = 90 nM) and its increased selectivity for LSD1 over MAO-A [54]. In 2015, Wu et al. designed and synthesized a series of 3-(piperidin-4-ylmethoxy) pyridine derivatives, all displaying potent inhibitory activity against LSD1 [55]. To unravel the potential inhibition mechanisms and binding modes, separate enzyme kinetics and molecular docking studies were conducted. Meanwhile, the experiments at the molecular and cellular levels were carried out to characterize the biological activities of these compounds. Particularly, compound **11** (K_i_ = 29 nM, compound **17**) [55] can increase H3K4 methylation in cells and strongly inhibit the proliferation of several leukemia and solid tumor cells. The molecular docking result (Figure 4B, left) revealed that compound **11** exhibited high inhibitory activity and well-docked to the substrate binding site of LSD1 with the lowest docking energy. This compound’s pyridine ring demonstrated favorable hydrophobic and electrostatic interactions with the flavin ring of FAD and residues Tyr761, Ala809, Thr810 and Ala539. Meanwhile, its 4-cyanophenyl group could establish good interactions with residues Ala539, Phe538, Trp695, His564 and Thr335. Notably, the protonated amine of the piperidin-4-ylmethoxy group established strong hydrogen bond and electrostatic interactions with Asp555, suggesting its pivotal role in LSD1 inhibition. Molecular docking substantiated their structure–activity relationship study, offering a clearer explanation for the differences in activity among compounds in this series. Based on the structure–activity relationship study by Wu et al., Wang et al. utilized 3D-QSAR (software: Sybyl-X 2.0), molecular docking and molecular dynamics simulation (software: Gromacs 5.1.4) techniques to investigate a series of 3-(piperidine-4-ylmethoxy) pyridine derivatives in 2018 [56]. 3D-QSAR is one of the most widely used methods in drug design. It establishes quantitative relationships between the structures of compounds (including their three-dimensional and electronic structures) and their biological effects (such as drug activity, toxicity, pharmacokinetic parameters and bioavailability) through a series of mathematical and statistical techniques. With these quantitative relationships, medicinal chemists can predict the biological activity of newly designed compounds, guiding drug design efforts and increasing the likelihood of success. Their 3D-QSAR model highlighted that pyridine derivatives’ interactions with LSD1 predominantly involved electrostatic, hydrophobic and hydrogen bond interactions, ensuring strong binding. The binding mode analysis based on molecular dynamics simulation (Figure 4B, right) showed that compound **11** was able to form stable hydrogen bonds with the LSD1 residues Asp555 and Lys661, respectively. Further energy decomposition results indicated that the interactions between compound **11** and residues Asp555, Lys661, Phe538, Leu693, Trp695 and Tyr761 were particularly important for the binding of this compound and LSD1. In the subsequent modification of compound **11** according to the theoretical results obtained, Wang et al. designed five new pyridine compounds (Figure 4C, compounds **12**–**16**) with better LSD1 inhibitory activities than compound **11**. The activity of these compounds on LSD1 needs to be further verified through experiments.

### 4.2. Pyrimidine Derivatives

**Figure 5:**
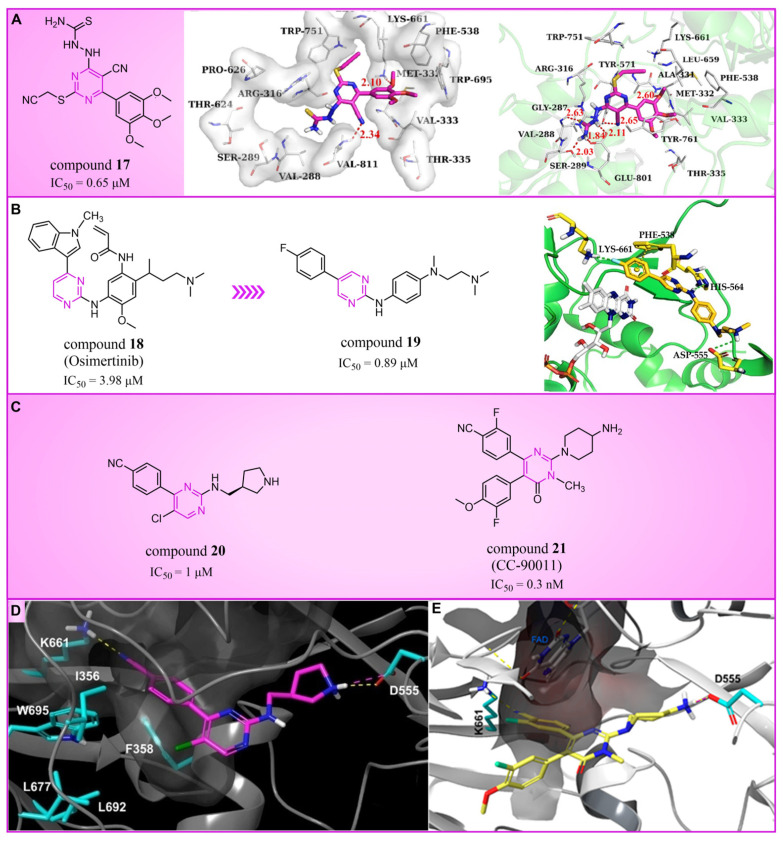
The structure of pyrimidine derivatives. (**A**) Compound **17** (**left**), the docking result ((**middle**), PDB code: 2H94) and the binding mode of compound **17** with LSD1 after MD simulation ((**right**), reprinted with permission from Ref. [64]. Copyright 2017 Elsevier); (**B**) compound **18** (Osimertinib) and compound **19,** and the binding mode of compound **19** with LSD1 (right, green dash lines represent the hydrogen bond interactions, yellow dash line is π–π stacking, reprinted with permission from Ref. [66]. Copyright 2022 Elsevier); (**C**) the structures of compound **20** and compound **21**; (**D**) the docking mode of compound **20** with LSD1 (adapted from Ref. [67]); (**E**) the co-crystal structure of compound **21** in complex with LSD1 (PDB code: 6W4K, reprinted from Ref. [67]).

Pyrimidine rings, extensively used in modern medicine [57,58,59], have garnered significant attention as MAO inhibitors [60,61,62]. In 2015, Liu’s team designed and synthesized a novel series of pyrimidine derivatives and evaluated their inhibitory properties against LSD1 [63]. In vitro, compound **17** (Figure 5A, compound **6b**, ${\mathrm{IC}}_{50}^{\mathrm{LSD}1}$ = 0.65 μM) [63] was found to be a better inhibitor in this series of compounds and showed strong cytotoxicity against gastric cancer cells with LSD1 overexpression. Furthermore, compound **17** had no significant inhibitory effect on MAO-A and MAO-B, indicating that this compound is a highly selective inhibitor for LSD1. In order to gain a deeper understanding of the interaction mechanism between these compounds and LSD1, Ding’s team investigated the structure–activity relationship of these pyrimidine derivatives and their binding modes with LSD1 using 3D-QSAR (software: Sybyl-X 2.0), molecular docking and molecular dynamics simulation techniques in 2017 [64]. The molecular docking results (software: MOE 2015.10, Figure 5A, middle) showed that compound **17** could bind to LSD1 well and had a similar binding mode to other compounds. The results of the molecular dynamics simulation for the LSD1/compound **17** complex system (Figure 5A, right) revealed that compound **17** was able to reach deep into the FAD binding pocket and formed a more stable structure with LSD1, with its aminothiourea moiety establishing crucial hydrogen bonds with residues Gly287, Ser289 and Glu801. Also, the cyano group of the compound formed hydrogen bond and electrostatic interactions with the residue Tyr571, and its 3-methoxy group formed a hydrogen bond with Ala331. Hydrophobic interactions between the 3,4,5-trimethoxyphenyl scaffold as well as the propargyl group and surrounding residues further contributed to the stability of the compound within the binding pocket. In 2019, Liu’s team discovered another pyrimidine analogue, compound **18** (Figure 5B, ${\mathrm{IC}}_{50}^{\mathrm{LSD}1}$ = 3.98 μM, Osimertinib) [65], whose molecular docking results showed that this compound occupied the FAD active site of LSD1 and could be used as a template to design novel pyrimidine derivatives. Hence, Wang et al. designed and synthesized a series of 2-aminopyrimidine derivatives based on compound **18** and investigated their inhibitory activities for LSD1 in 2022 [66]. In particular, compound **19** (Figure 5B, ${\mathrm{IC}}_{50}^{\mathrm{LSD}1}$ = 0.89 μM, compound **X43**) exhibited significant inhibitory activity for LSD1 at both the molecular and cellular levels and showed significant selectivity for LSD1 compared to MAO-A/B (>50-fold). Molecular docking (Figure 5B, right) unveiled hydrogen bonds between compound **19** and residues Asp555, His564 and Lys661, contributing to its effective inhibition. Hydrophobic interactions also played a role in maintaining the compound’s stability within the binding pocket. In 2020, Kanouni and other researchers conducted a high-throughput screening for a diversity library containing 300,000 compounds and a smaller structure-based design library [67]. High-throughput screening is a strategy for screening large, complex libraries quickly and effectively. It allows chemical libraries to be screened rapidly and cost-effectively to identify the most promising compounds with activity against specific biological targets. Based on the results of molecular docking and cell experiments, they chose to perform a series of modifications and optimizations on compound **20** (Figure 5C, ${\mathrm{IC}}_{50}^{\mathrm{LSD}1}$ = 1 μM, compound **1**). Ultimately, they successfully developed a compound, compound **21** (Figure 5C, ${\mathrm{IC}}_{50}^{\mathrm{LSD}1}$ = 0.3 nM, CC-90011), which exhibited significant inhibitory activity against LSD1 [67]. Molecular docking (Figure 5D) highlighted interactions between the benzonitrile of compound **20** and LSD1 residue Lys661, with the pyrrole ring forming a hydrogen bond and a salt bridge with Asp555. With inspiration drawn from the structure–activity relationship and ADME properties of compound **20** and its derivatives, they designed compound **21** and obtained the X-ray co-crystal structure of compound **21** in the AOL pocket of LSD1 (Figure 5E, PDB code: 6W4K). This compound has been confirmed to be a highly effective reversible inhibitor of LSD1, showing effective targeted induction of cell differentiation in acute myeloid leukemia (AML) and small-cell lung cancer (SCLC) cell lines, and demonstrating anti-tumor efficacy in patient-derived xenograft (PDX) SCLC models. It has now entered phase II trials for the treatment of SCLC. The success of compound **21** highlights the great potential of Pyrimidine series inhibitors as LSD1 drugs. With the further study of the mechanism of interaction between pyrimidine compounds and LSD1 and the optimization of its structure, it is expected to develop new anti-tumor drugs with excellent properties.

### 4.3. Azole Derivatives

#### 4.3.1. Triazole Derivatives

**Figure 6:**
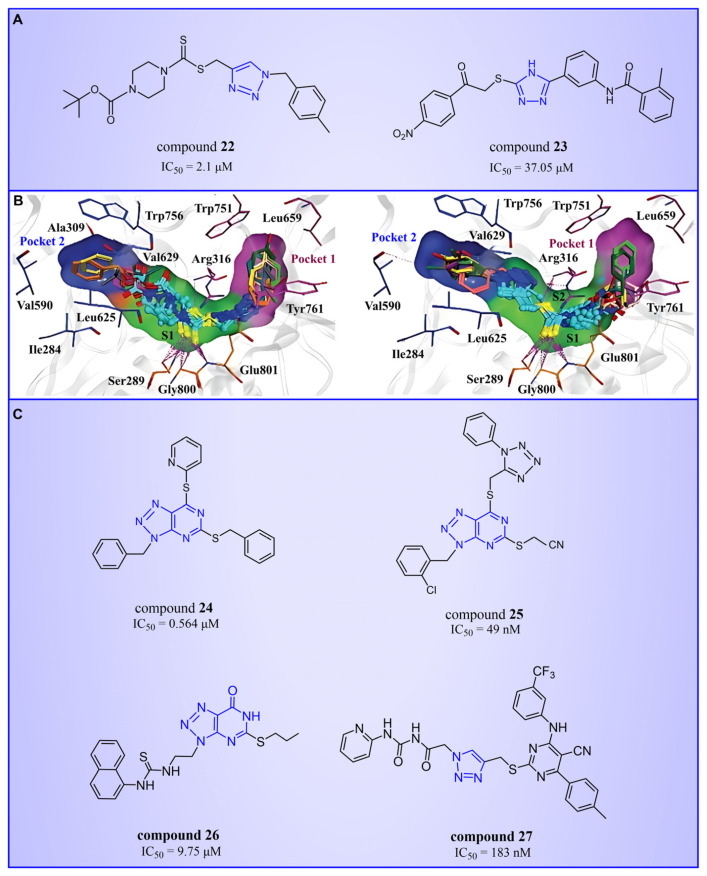
The structures of triazole derivatives (**A**) compounds **22** and **23**; (**B**) two conformations of triazole derivatives at the binding site (type A, the triazole moiety in close proximity to pocket 1, (**left**); type B, the triazole moiety in close proximity to pocket 2, (**right**), reprinted with permission from Ref. [73]. Copyright 2018 Royal Society of Chemistry); (**C**) compounds **24**–**27**.

**Figure 7:**
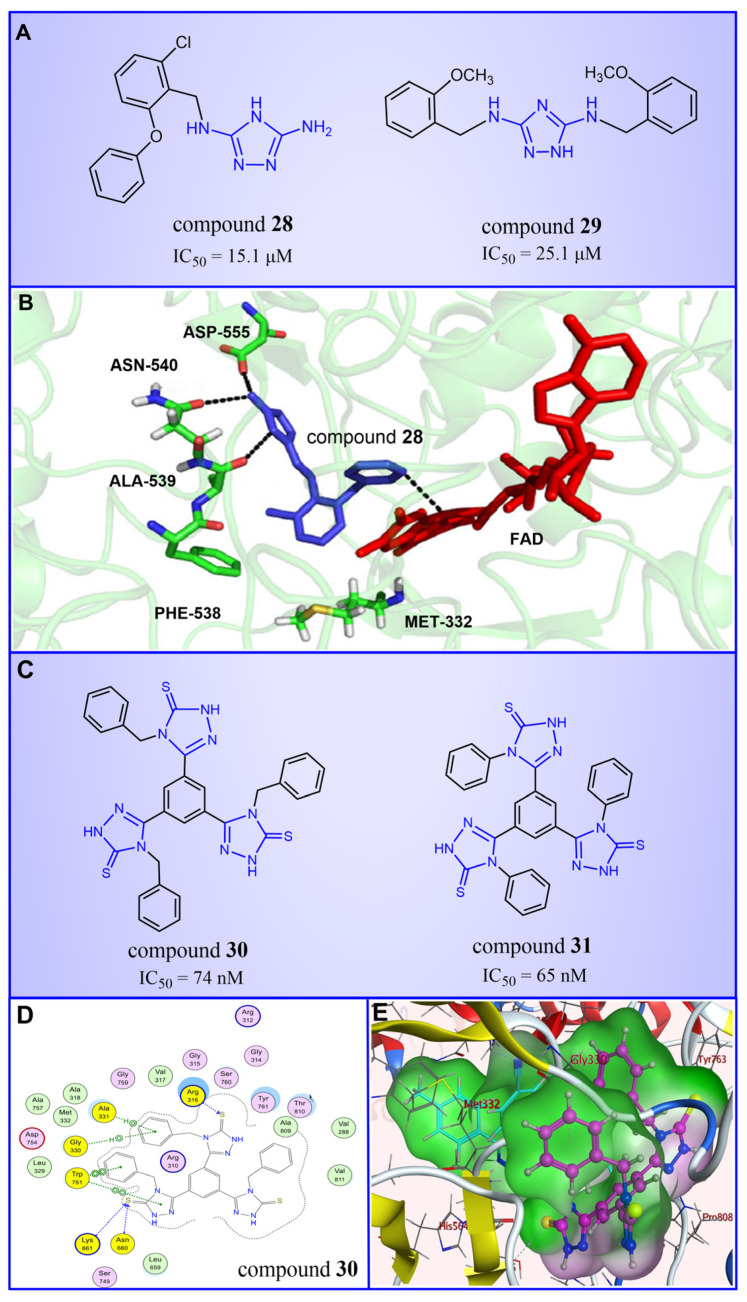
The structures of triazole derivatives. (**A**) Compounds **28** and **29**. (**B**) Complex structure of LSD1 upon binding to compound **28** (PDB code: 3ZMT, reprinted with permission from Ref. [79]. Copyright 2018 Royal Society of Chemistry); the key amino acids are illustrated. (**C**) Compounds **30** and **31**; (**D**) 2D diagram of the interaction between compound **30** and LSD1 (reprinted from Ref. [80]); (**E**) surface map for the compound **30** inside active site (reprinted from Ref. [80]).

Triazole compounds, distinguished by their high activity, low toxicity, favorable bioavailability and exceptional pharmacokinetic properties, have emerged as a prominent focus in drug development [68,69,70]. In 2013, Zheng et al. strategically designed and synthesized a series of 1,2,3-triazole-dithiocarbamate hybrids, showcasing compound **22** (Figure 6A, ${\mathrm{IC}}_{50}^{\mathrm{LSD}1}$ = 2.1 μM, compound **26**) as a lead, with potent LSD1 inhibitory effects and superior selectivity over MAO-A (${\mathrm{IC}}_{50}^{\mathrm{MAO}\text{-}A}$ > 1250 μM) and MAO-B (${\mathrm{IC}}_{50}^{\mathrm{MAO}\text{-}B}$ > 1250 μM) [71,72]. Notably, compounds containing a tert-butyloxycarbonyl group exhibited enhanced inhibitory activity on LSD1. In order to investigate the binding modes between these 1,2,3-triazole-dithiocarbamate hybrids and LSD1, Sun et al. from the same group performed a further study using computational methods such as molecular docking, molecular dynamics simulation and steered molecular dynamics (SMD) simulation in 2018 [73]. SMD simulation is an important branch of molecular dynamics which belongs to the category of non-equilibrium dynamics simulation. The main goal of SMD simulation is to study the dynamic processes of ligand binding and unbinding in complex systems. By applying external forces, SMD can simulate force-directed processes in biological molecules or chemical reactions, thereby providing insight into the structural and functional relationships of biological molecules. They found that these small molecules presented two conformations with completely opposite directions at the binding pocket of the FAD region (named type A and type B, as shown in Figure 6B). Interestingly, in both binding modes, this series of inhibitors could form hydrogen bonds with the residues Arg316, Gly800 and Ser289. Meanwhile, the residue Trp756 at the pocket’s exit played a pivotal role due to steric hindrance and its ability to engage in hydrophobic interactions with inhibitors. In addition, the type B conformation was predicted to be a more favorable and stable binding mode for these inhibitors with LSD1. In the same year, Sun et al. developed a pharmacophore model based on triazole-dithiocarbamate compounds and performed a virtual screening of the SPECS database to develop novel LSD1 inhibitors [74]. This method is a commonly used approach in drug design and prediction. Virtual screening is to screen a large number of compounds using a computer to predict the interaction and affinity between compounds and target proteins, so that compounds with potential biological activities can be screened quickly and efficiently. In general, pharmacophore models can serve as a constraint in virtual screening. It involves creating an accurate and predictive model based on known pharmacophore information and the structures of active compounds to identify and validate compounds with similar pharmacophores, thereby screening for compounds that may have similar biological activities. As a result, this method can rapidly and accurately predict the activity of candidate compounds in the early stages of drug design, ultimately accelerating the process of drug discovery and development. Compound **23** (Figure 6A, ${\mathrm{IC}}_{50}^{\mathrm{LSD}1}$ = 37.05 μM, compound **1**) exhibited the highest similarity to template compound **22**, demonstrating notable inhibitory activity against LSD1. From 2017 to 2020, the group extended their efforts to synthesize a series of 1,2,3-triazole-fused pyrimidine derivatives through virtual screening and structural optimization, with compounds **24**–**27** (Figure 6C) exhibiting significant LSD1 inhibitory activities [75,76,77,78]. This suggests that triazole-fused pyrimidine could be a dominant scaffold for designing novel LSD1 inhibitors. Furthermore, Kutz et al. obtained compound **28** (Figure 7A, ${\mathrm{IC}}_{50}^{\mathrm{LSD}1}$ = 15.1 μM, compound **6**) and compound **29** (Figure 7A, ${\mathrm{IC}}_{50}^{\mathrm{LSD}1}$ = 25.1 μM, compound **7**) [79] with 3,5-diamino-1,2,4-triazoles as a new scaffold through the virtual screening of the Maybridge Hitfinder 5 compound library. As shown in Figure 7B, the result of molecular docking between compound **28** and LSD1 showed that the compound could bind tightly to the active site of the substrate region and was able to form hydrogen bonds with Asn540, Asp555 and Ala539, as well as π–π stacking interactions with the flavin ring of FAD. In 2022, Alsehli et al. designed and synthesized a series of 1,2,4-triazole derivatives, with compounds **30** (Figure 7C, ${\mathrm{IC}}_{50}^{\mathrm{LSD}1}$ = 74 nM, compound **22**) and **31** (Figure 7C, ${\mathrm{IC}}_{50}^{\mathrm{LSD}1}$ = 65 nM, compound **23**) displaying exceptional inhibitory activity against LSD1 [80]. In particular, the molecular docking results (software: MOE 2014, Figure 7D) showed that compound **30** could form hydrogen bonds with residues Arg316, Asn660 and Lys661, respectively, while one of the aromatic rings linked to the triazole ring could also form π–π stacking interactions with Trp751. Additionally, the compound could form arene–H interactions with Gly330 and Ala331, respectively, thus maintaining good stacking against the hydrophobic surface of the protein. From the hydrophobic surface map (Figure 7E), it could be found that compound **30** was highly lipophilic and was able to make more contact with the lipophilic pocket of LSD1, thus maintaining a stable binding of the two. These interaction details can serve as theoretical foundations and guidance for the future design of such compounds.

#### 4.3.2. Thiazole Derivatives

**Figure 8:**
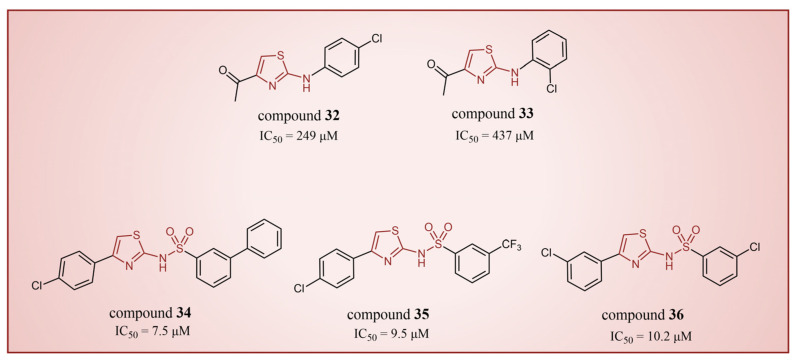
The structures of thiazole derivatives, compounds **32**–**36**.

**Figure 9:**
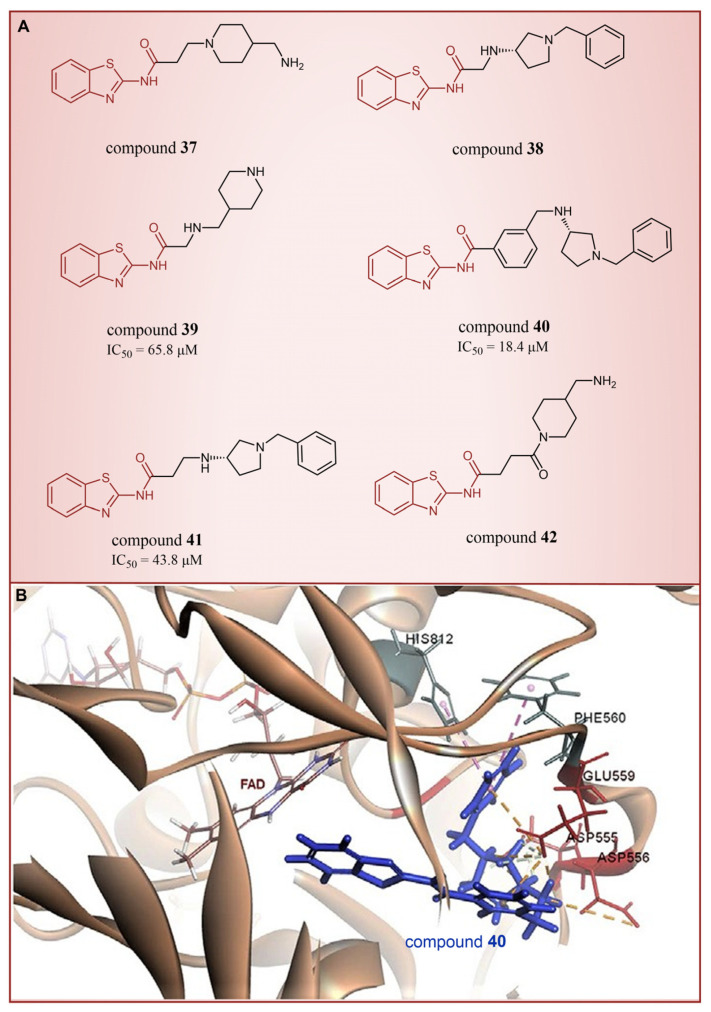
The structures of thiazole derivatives. (**A**) Compounds **37**–**42**; (**B**) compound **40** bound inside the active site of LSD1; yellow dotted line represents electrostatic interactions; pink dotted lines are π–π stacking (reprinted with permission from Ref. [82]. Copyright 2019 Elsevier).

In 2013, Hitchin et al. conducted a comprehensive screening of a fragment library comprising 2466 compounds using biochemical fragment screening technology. From this, two aminothiazole compounds, namely compound **32** (Figure 8, ${\mathrm{IC}}_{50}^{\mathrm{LSD}1}$ = 249 μM, compound **7**) and compound **33** (Figure 8, ${\mathrm{IC}}_{50}^{\mathrm{LSD}1}$ = 437 μM, compound **8**), were identified [54]. Subsequently, these two compounds underwent a systematic process, leading to the generation of a series of aminothiazole and thiazolesulfonamide derivatives. In particular, compound **34** (Figure 8, ${\mathrm{IC}}_{50}^{\mathrm{LSD}1}$ = 7.5 μM, compound **16k**), compound **35** (Figure 8, ${\mathrm{IC}}_{50}^{\mathrm{LSD}1}$ = 9.5 μM, compound **16q**) and compound **36** (Figure 8, ${\mathrm{IC}}_{50}^{\mathrm{LSD}1}$ = 10.2 μM, compound **19c**) exhibited significant inhibitory effects on LSD1. To elucidate the intricate binding modes between these derivatives and LSD1, Maltarollo et al. constructed 2D and 3D QSAR models based on 54 aminothiazole and thiazolesulfonamide derivatives using a holographic quantitative structure–activity relationship (HQSAR) and comparative molecular interaction field analysis (CoMFA) in 2015 [81]. The results revealed that compounds containing bulky and aromatic substituents at the thiazole ring could form favorable steric and electrostatic interactions with the residues at the LSD1 active site. Notably, the thiazolesulfonamide group emerged as a crucial contributor to the strong biological activity exhibited by thiazolesulfonamide derivatives. Therefore, in the quest for designing highly potent LSD1 inhibitors, the thiazolesulfonamide group merits careful consideration as a pivotal functional group. In 2019, Alnabulsi et al. screened the Maybridge Ro3 2000 Diversity Fragments Library with a fragment-based drug design method, and obtained two groups of chemically diverse fragments [82]. Based on these fragments, 24 compounds with drug-like properties were designed using the fragment growth approach, followed by molecular docking simulations. The docking results and binding mode analyses led to the identification of six promising compounds (Figure 9A, compounds **37**–**42**) featuring an amino-carboxamidebenzothiazole scaffold. Subsequent biological evaluations revealed that compound **40** (${\mathrm{IC}}_{50}^{\mathrm{LSD}1}$ = 18.4 μM) exhibited commendable inhibitory activity against LSD1, forming π–π stacking with FAD and establishing electrostatic interactions with Asp555 and Asp556 (Figure 9B). Currently, the team is actively optimizing compound **40** in pursuit of designing more efficacious LSD1 inhibitors.

#### 4.3.3. Pyrazole Derivatives

**Figure 10:**
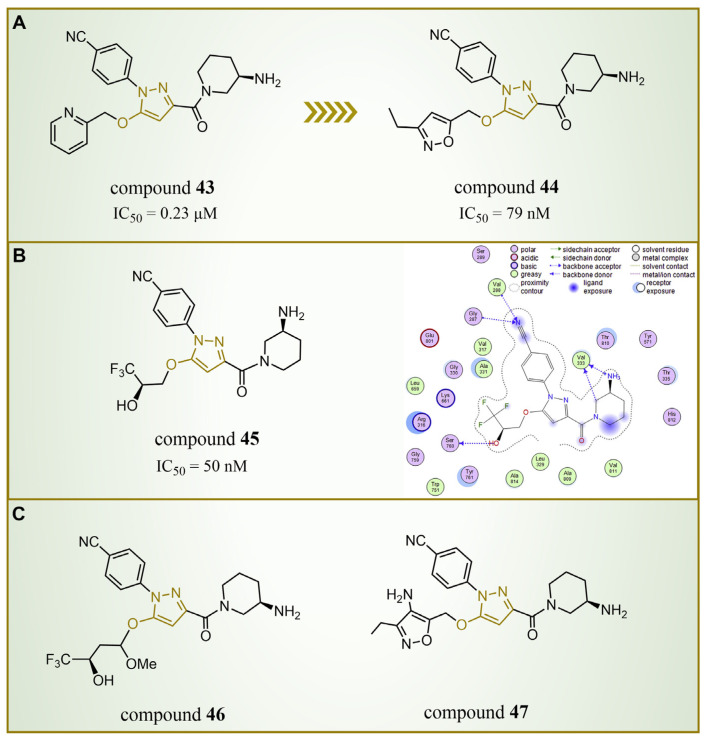
The structures of pyrazole derivatives. (**A**) Compounds **43** and **44**; (**B**) compound **45,** along with a 2D diagram depicting its interaction with LSD1 (reprinted with permission from Ref. [85]. Copyright 2019 Elsevier); (**C**) compounds **46** and **47**.

In 2015, Chen’s group introduced compound **43** (Figure 10A, ${\mathrm{IC}}_{50}^{\mathrm{LSD}1}$ = 0.23 μM, compound **44**), showcasing remarkable LSD1 inhibitory activity in vitro [83]. Building upon this success, Mould et al. designed and synthesized a series of LSD1 reversible inhibitors with a 5-hydroxypyrazole structure in 2017 [84]. Rigorous assessments, including surface plasmon resonance (SPR) and biochemical analyses, identified compound **44** (Figure 10A, ${\mathrm{IC}}_{50}^{\mathrm{LSD}1}$ = 79 nM, compound **11p**) [84] as the most potent LSD1 inhibitor, boasting exceptional bioavailability. In 2018, Seraj et al. comprehensively explored the inhibition mechanisms of this series of compounds and the correlation between the 5-hydroxypyrazole structure and its inhibitory activity by employing molecular docking, classification and QSAR modeling techniques [85]. A detailed examination of the docking result involving compound **45** (software: MOE 2014.09, Figure 10B, ${\mathrm{IC}}_{50}^{\mathrm{LSD}1}$ = 50 nM, compound **7**) and LSD1 affirmed the pivotal role of residues Arg316, Thr810 and Tyr761 in the inhibition mechanism (Figure 10B), engaging in robust polar interactions with the inhibitors. Furthermore, compound **45** established hydrogen bond interactions with Gly287, Val288, Val333 and Ser760, fortifying the bond with LSD1 and thereby enhancing the inhibitory effects. The revelation of these intricate interactions not only enhances our understanding of the inhibition mechanism, but also provides crucial insights for refining future inhibitor designs. Meanwhile, the design of six new 5-hydroxypyrazole analogues was undertaken, with their activities predicted based on the developed QSAR models. Notably, compound **46** (Figure 10C, compound **new 1**) and compound **47** (Figure 10C, compound **new 3**) surpassed compound **45** in both the predicted activity and docking score, hinting at their potential as promising candidates for further exploration.

In general, the research on azole compounds (including triazole, thiazole and pyrazole) as LSD1 inhibitors has attracted extensive attention in recent years. A large number of synthesis, screening and structural optimization studies have led to a series of azole compounds with good LSD1 inhibitory activity, such as compounds **25, 34** and **35**. Subsequent further research based on these compounds is to be expected.

### 4.4. Thieno[3,2-b]pyrrole Derivatives

**Figure 11:**
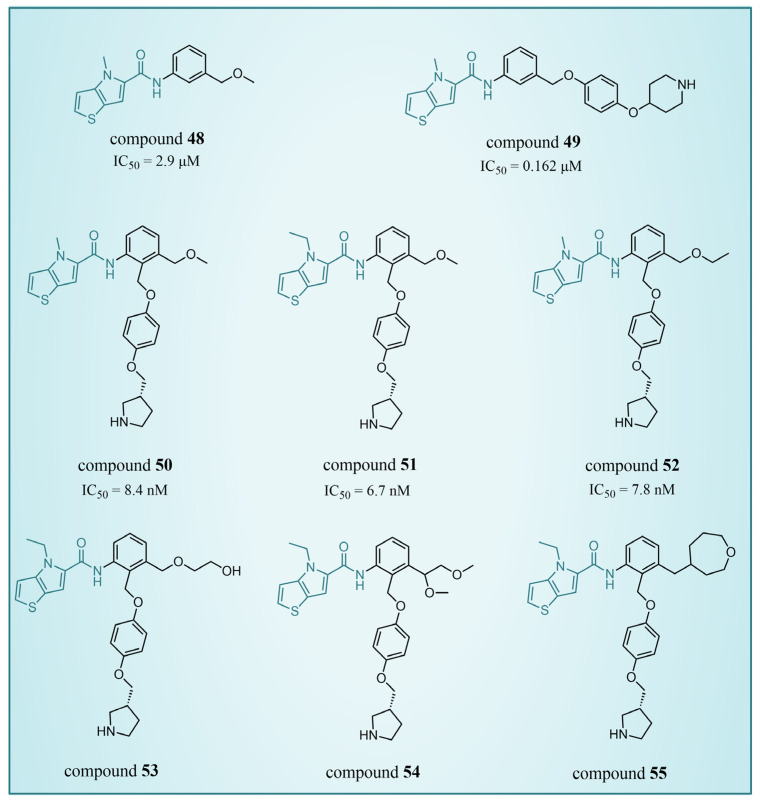
The structures of thieno[3,2-b]pyrrole derivatives, compounds **48**–**52**.

**Figure 12:**
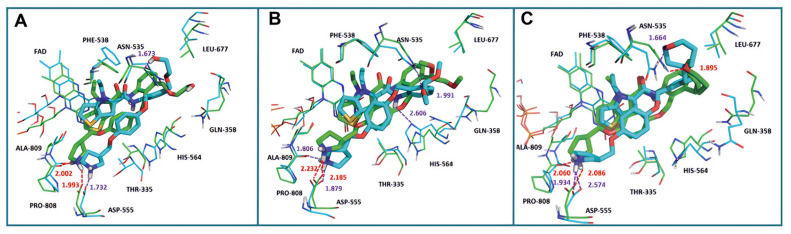
The superposition of the docking structures (green) and MD average structures (cyan) of LSD1 with (**A**) compound **53**, (**B**) compound **54** and (**C**) compound **55**, respectively (reprinted from Ref. [88]).

In 2017, Sartori’s team identified compound **48** (Figure 11, ${\mathrm{IC}}_{50}^{\mathrm{LSD}1}$ = 2.9 μM, compound **19**) via the high-throughput screening of a bioactive compound library containing 34,000 compounds, based on *N*-phenyl-4*H*-thieno[3,2-b]pyrrole-5-carboxamide [86]. This compound showcased LSD1 inhibitory activity by competitively interacting with its substrate. Subsequent optimization led to the development of compound **49** (Figure 11, ${\mathrm{IC}}_{50}^{\mathrm{LSD}1}$ = 0.162 μM, compound **90**), demonstrating superior inhibitory effects compared to its precursor. Expanding on the thieno[3,2-b]pyrrole scaffold, the team designed a series of thieno[3,2-b]pyrrole-5-carboxamides in the same year, analyzing their structure–activity relationships. During this process, a crucial molecular feature, ortho substitution in the benzamide, was identified [87]. Particularly, compounds **50** (Figure 11, ${\mathrm{IC}}_{50}^{\mathrm{LSD}1}$ = 8.4 nM, compound **46**), **51** (Figure 11, ${\mathrm{IC}}_{50}^{\mathrm{LSD}1}$ = 6.7 nM, compound **49**) and **52** (Figure 11, ${\mathrm{IC}}_{50}^{\mathrm{LSD}1}$ = 7.8 nM, compound **50**) exhibited significant inhibitory activities against LSD1, stimulating further exploration into thieno[3,2-b]pyrrole compounds. In 2020, our group employed 3D-QSAR, molecular docking and molecular dynamics simulations to delve into the binding modes of these compounds with LSD1 [88]. Guided by the structure–activity relationships gleaned from the 3D-QSAR model, we designed eight candidate compounds. Among them, compounds **53**–**55** (Figure 11, compounds D4, D5 and D8) emerged as promising LSD1 inhibitors, surpassing the activity of compound **51**. The binding model map (Figure 12) illustrated that compounds **53**–**55** formed hydrogen bonds with Asn535, Asp555 and Pro808. Differently, compound **54** formed a unique hydrogen bond with residue His564. An energy decomposition analysis revealed that residues Val333, Phe538, Leu677, Trp695, Thr761 and FAD contributed to stabilizing these inhibitors at the substrate binding sites. To assess the synthetic feasibility and address potential issues related to metabolism and toxicity, we conducted ADME prediction and bioavailability analysis for the newly designed compounds **53**–**55**, as well as reference compound **51**. The results indicated that the novel designed compounds might serve as safer and more active LSD1 inhibitors. Our research will provide crucial insights for future designs. In the same year, Zhang et al. undertook similar research, establishing a 3D-QSAR model using Discovery Studio 3.0 and employing molecular docking simulations to explore possible binding modes between inhibitors and LSD1 [89]. The calculated binding free energy using the MM/GBSA method aligned well with experimental biological activities. Through the optimization of compound **52**, they designed six new LSD1 inhibitors, predicting their activities with the 3D-QSAR model. The results indicated that all the newly designed compounds exhibited superior inhibitory activity compared with compound **52**. These collective efforts contribute valuable knowledge to the ongoing quest for effective LSD1 inhibitors.

### 4.5. Indole Derivatives

**Figure 13:**
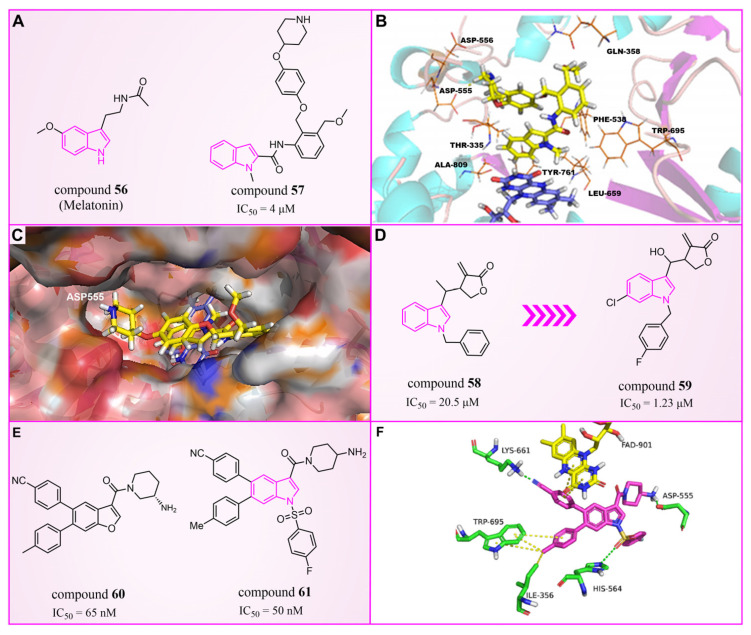
The structures of indole derivatives. (**A**) Compounds **56** and **57**; (**B**) complex structure of LSD1 upon binding to compound **57**; the key residues are labeled (reprinted with permission from Ref. [94]. Copyright 2018 Elsevier). (**C**) Compound **57** in the pocket cavity of LSD1 (reprinted with permission from Ref. [94]. Copyright 2018 Elsevier). (**D**) Compounds **58** and **59**; (**E**) compounds **60** and **61**; (**F**) complex structure of LSD1 upon binding to compound **61** (PDB code: 5YJB); green dash lines represent hydrogen bond interactions; yellow dash lines are π–π stacking (reprinted with permission from Ref. [96]. Copyright 2022 Elsevier).

The indole ring, with its significant chemical properties and pharmacological effects, has emerged as a crucial scaffold in the exploration of anticancer drugs, particularly as scientists investigate LSD1 inhibitors [90,91,92]. In 2017, Yang et al. found that compound **56** (Figure 13A, melatonin), featuring the indole skeleton, hindered the proliferation of oral cancer cells by suppressing LSD1 overexpression [93]. Expanding on this, in 2018, Xi et al. designed and synthesized a series of 4-(4 benzyloxy) phenoxypiperidine compounds that could be used as LSD1 reversible inhibitors, among which compound **57** (Figure 13A, ${\mathrm{IC}}_{50}^{\mathrm{LSD}1}$ = 4 μM, compound **10d**), containing an indole moiety, showed significant inhibitory activity against LSD1 and high selectivity relative to MAO-A (${\mathrm{IC}}_{50}^{\mathrm{MAO}\text{-}A}$ = 71 μM) and MAO-B (${\mathrm{IC}}_{50}^{\mathrm{MAO}\text{-}B}$ = 138 μM) [94]. Molecular docking results (Figure 13B,C) illustrated that compound **57** occupied the substrate region of LSD1 in a U-shaped conformation. Meanwhile, a hydrogen bond formed between the N atom at the end of the molecule and the residue Asp555, and the indole ring formed π–π stacking with FAD. In 2019, Liu’s team identified an interesting indole derivative, compound **58** (Figure 13D, ${\mathrm{IC}}_{50}^{\mathrm{LSD}1}$ = 20.5 μM, compound **1a**), containing butanolide, through an initial screening of their internal compound library [95]. Subsequently, they optimized the structure of compound **58**, designed and synthesized a series of new indole compounds, and studied the structure–activity relationship of these compounds. They found that the phenyl groups of the compounds and the tetrahedral configuration of the link atom to N1 from phenyl groups were essential to their activities. In particular, compound **59** (Figure 13D, ${\mathrm{IC}}_{50}^{\mathrm{LSD}1}$ = 1.23 μM, compound **9e**), containing 5-chloro and *N*-(4-*F*-benzyl) indole, was proven to be an effective and irreversible LSD1 inhibitor. While the binding mode between these compounds and LSD1 remains unclear, future computational simulations can shed light on their inhibitory mechanisms. This year, Zhang et al. achieved remarkable success by replacing the benzofuran ring with an indole ring in compound **60** (Figure 13E, ${\mathrm{IC}}_{50}^{\mathrm{LSD}1}$ = 65 nM, compound **17i**), leading to the design of compound **61** (Figure 13E, ${\mathrm{IC}}_{50}^{\mathrm{LSD}1}$ = 50 nM, compound **B35**), with outstanding inhibitory activity against LSD1 and a stable metabolism [96]. In agreement with the experimental results, the docking results (Figure 13F) highlighted that compound **61** exhibited the best docking score, forming hydrogen bond interactions with residues Trp552, Asp555, His564 and Lys661. Moreover, compound **61** maintained stability in the lipophilic active pocket through hydrophobic interactions with residues Met332, Val333, Ile356, Ala539, Trp695, Ala809 and FAD. Unfortunately, in the prediction of pharmacokinetic characteristics, the compound exhibited poor oral bioavailability. Consequently, there exists the potential to augment the oral bioavailability of the compound through structural optimization, thereby facilitating the design and synthesis of novel LSD1 inhibitors. In summary, indole compounds, as inhibitors of LSD1, present expansive research prospects and potential therapeutic applications. Nonetheless, ongoing investigations into their utility as LSD1 inhibitors are still in their nascent stages, necessitating more comprehensive studies to fully elucidate their mechanism of action and efficacy. Furthermore, in subsequent research endeavors, it is imperative to assess their pharmacokinetic characteristics and bioavailability. This evaluation, coupled with ensuring their robust LSD1 inhibitory activity, is vital to ascertain favorable drug properties and therapeutic effects.

### 4.6. Quinoline Derivatives

**Figure 14:**
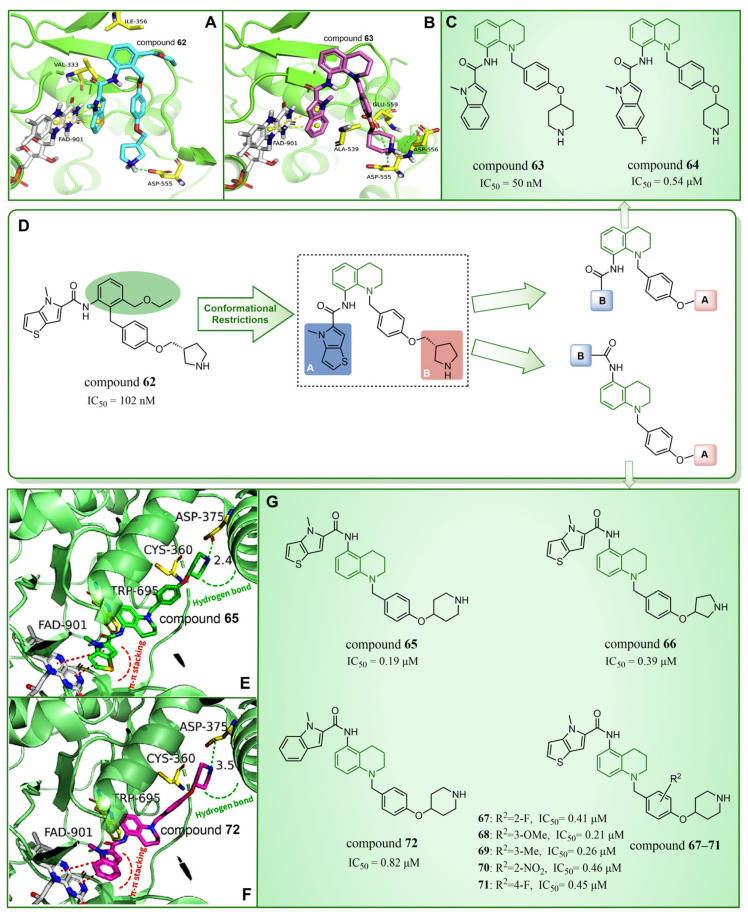
The structures and binding mode analysis of quinoline derivatives. (**A**) Binding mode of LSD1 with compound **62** (reprinted with permission from Ref. [97]. Copyright 2020 Elsevier); (**B**) binding mode of LSD1 with compound **63** (reprinted with permission from Ref. [97]. Copyright 2020 Elsevier); (**C**) compound **63** and compound **64**; (**D**) compound **62** and design and modification strategy of the target compound; (**E**) the proposed binding mode of LSD1 with compound **65** (reprinted with permission from Ref. [98]. Copyright 2021 John Wiley and Sons); (**F**) the proposed binding mode of LSD1 with compound **72** (reprinted with permission from Ref. [98]. Copyright 2021 John Wiley and Sons); (**G**) compounds **65**–**72**.

In 2020, Wang et al. took LSD1 reversible inhibitor **62** (Figure 14D, ${\mathrm{IC}}_{50}^{\mathrm{LSD}1}$ = 7.8 nM, compound **8**) [87] as a lead compound and made significant discoveries through continuous structural optimization research, leading to the identification of a tetrahydroquinoline scaffold with excellent LSD1 inhibitory activity [97]. Subsequently, they developed a series of tetrahydroquinoline derivatives based on this scaffold. Experimental study on enzyme activity inhibition and in vivo pharmacodynamic evaluation revealed that compounds **63** (Figure 14C, ${\mathrm{IC}}_{50}^{\mathrm{LSD}1}$ = 50 nM, compound **18s**) and **64** (Figure 14C, ${\mathrm{IC}}_{50}^{\mathrm{LSD}1}$ = 0.54 μM, compound **18x**) exhibited excellent drug properties. The molecular docking results (software: Glide 9.7, Figure 14A,B) illustrated the stable binding of this compound series in a U-shaped conformation within the substrate binding site. Particularly, crucial π–π stacking interactions were observed between FAD and the thieno[3,2-b]pyrrole fragment of compound **62**, as well as the indole ring of compound **63**, underscoring their significance in mediating inhibitory activities. The difference lies in the fact that compound **62** was surrounded by Val333 and Ile356, forming hydrophobic interactions with these residues, while compound **63** did not. However, there was a rich hydrogen bond interaction network between compound **63** and residues Asp555, Asp556 and Glu559, which slightly enhanced its activity compared to compound **62**. In the subsequent year, Yan et al. from the same group utilized conformational restriction and fragment growth methods to design and synthesize 41 new 5-aminotetrahydroquinoline LSD1 inhibitors acting on Asp375, and performed structure–activity relationship studies, molecular docking studies and the prediction of ADME properties [98]. Among them, compounds **65**–**72** (Figure 14G, compounds **A6**, **A8**, **B1**–**B5** and **C4**) demonstrated favorable inhibitory effects on LSD1. From the molecular docking results (Figure 14E,F), it was known that all compounds in this series were docked to the substrate binding pocket and had similar binding modes. Importantly, the nitrogen atom at the end of each compound formed a hydrogen bond with the residue Asp375. In particular, the pyrrole rings of compounds **65** and **72** could form strong π–π stacking interactions with FAD. The tetrahydroquinoline group formed a hydrophobic interaction with Trp695, and the attached benzene ring formed a hydrophobic interaction with Cys360. Notably, compound **65** was closer to Asp375, which may be the reason why the activity of **65** was higher than that of **72**. However, the predicted ADME properties of compounds **65**–**72** were not satisfactory, and they were far from the properties of ideal drugs. In the next step, it is necessary to optimize the physicochemical properties of these compounds to improve their drug-like properties. In summary, quinoline is a highly promising scaffold for designing efficient LSD1 inhibitors. Its consistent demonstration of excellent inhibitory activity, coupled with ongoing structural optimization and molecular design, positions it as a robust foundation for the development of potent LSD1 inhibitors. Continued research and exploration are anticipated to unveil innovative quinoline derivatives with potential applications in cancer therapy and beyond.

### 4.7. Phenyloxazole Derivatives

**Figure 15:**
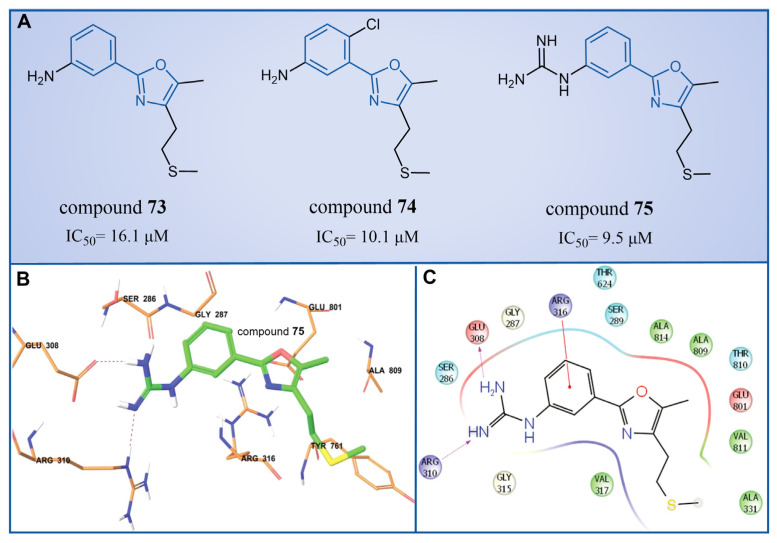
(**A**) The structures of phenyloxazole derivatives compounds **73**–**75**; (**B**) binding orientation and (**C**) 2D diagram of interactions of compound **75** at the LSD1 binding site (reprinted with permission from Ref. [99]. Copyright 2013 Royal Society of Chemistry).

In 2013, Dulla et al. [99] innovatively designed a series of phenyloxazole derivatives as reversible inhibitors of LSD1. They connected the key pharmacophore characteristics of MAO-, polyamine/guanidine and methionine-based peptide inhibitors through the use of oxazole groups, and anticipated the docking sites, interaction pattern and theoretical feasibility of small molecules of LSD1. A pharmacological analysis demonstrated that compounds **73**–**75** (Figure 15A, compounds **6a**, **6b** and **9a**, ${\mathrm{IC}}_{50}^{\mathrm{LSD}1}$ = 16.1, 10.1 and 9.5 μM, respectively) exhibited inhibitory activities in in vitro (${\mathrm{IC}}_{50}^{\mathrm{HeLacel}1}$ = 1.48 nM), cell culture and in vivo systems. Furthermore, molecular modeling techniques were employed to investigate the binding modes and stabilities of these three compounds with LSD1. As shown in Figure 15B, the guanidine group of compound **75** formed hydrogen bonds with Glu308 and Arg310, and the benzene ring formed a π–cation interaction with Arg316. The molecular dynamics analysis revealed that the protein–ligand complex maintained its stability, with the ligand consistently bound in the anticipated orientation. This pioneering research not only expanded the repertoire of structural classes of LSD1 inhibitors, but also laid the groundwork for future explorations into this category of inhibitors.

## 5. Natural Products

### 5.1. Sanguinarine

**Figure 16:**
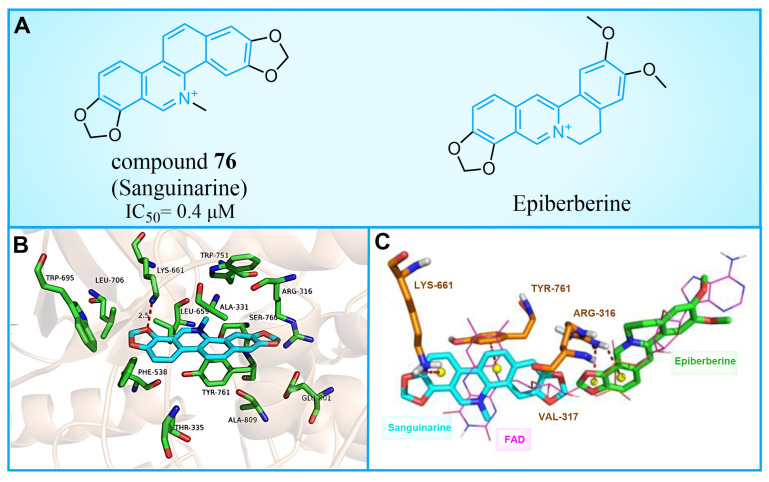
(**A**) The structure of compound **76** (Sanguinarine) and Epiberberine; (**B**) predicted binding mode of compound **76** in the active site of LSD1 (PDB: 2V1D, reprinted from Ref. [100]); (**C**) overlap of the binding poses of compound **76**, Epiberberine and FAD (reprinted from Ref. [100]).

Compound **76** (Figure 16A, Sanguinarine) is a natural alkaloid with a polycyclic skeleton of benzophenanthridine, derived primarily from poppy fumaria species. Qin et al. found that compound **76** could effectively inhibit LSD1 (${\mathrm{IC}}_{50}^{\mathrm{LSD}1}$ = 0.4 μM) by screening their internal natural compound library, and this inhibition was reversible [100]. In H1299 and H1975 cells, compound **76** inhibited the demethylation of LSD1, which confirmed its cellular activity to the enzyme. Further research showed that compound **76** has a strong ability to inhibit colony formation, migration and invasion, and induce the apoptosis of H1299 and H1975 cells. By employing molecular docking techniques, the binding pattern of compound **76** to LSD1 was investigated (Figure 16B). Compound **76** demonstrated hydrophobic interactions with Tyr761, Leu659, Lys661, Thr335, Ala809 and Val811 within the binding pocket. It further formed a hydrogen bond with Lys661, in addition to a π–π stacking interaction with Tyr761 and Leu659. As illustrated in Figure 16B, compound **76** and pyrido [3,4-b] quinoxaline of FAD exhibit significant overlap, suggesting that sanguinarine could disable LSD1 by competitively binding to FAD within the binding pocket of LSD1. Conversely, Epiberberine (Figure 16A, ${\mathrm{IC}}_{50}^{\mathrm{LSD}1}$ = 0.14 μM), featuring an isoquinoline tetracyclic scaffold [101], was found to bind to another region of the FAD pocket (Figure 16C). This provides evidence for the impact of a similar structure with differing substituents on inhibitory activity. In view of the general antibacterial and anti-inflammatory properties of natural products [102,103], the four-ring scaffold structure based on compound **76** could be further optimized to obtain high-efficiency and low-toxicity candidate drug molecules.

### 5.2. Phenolic Compounds

**Figure 17:**
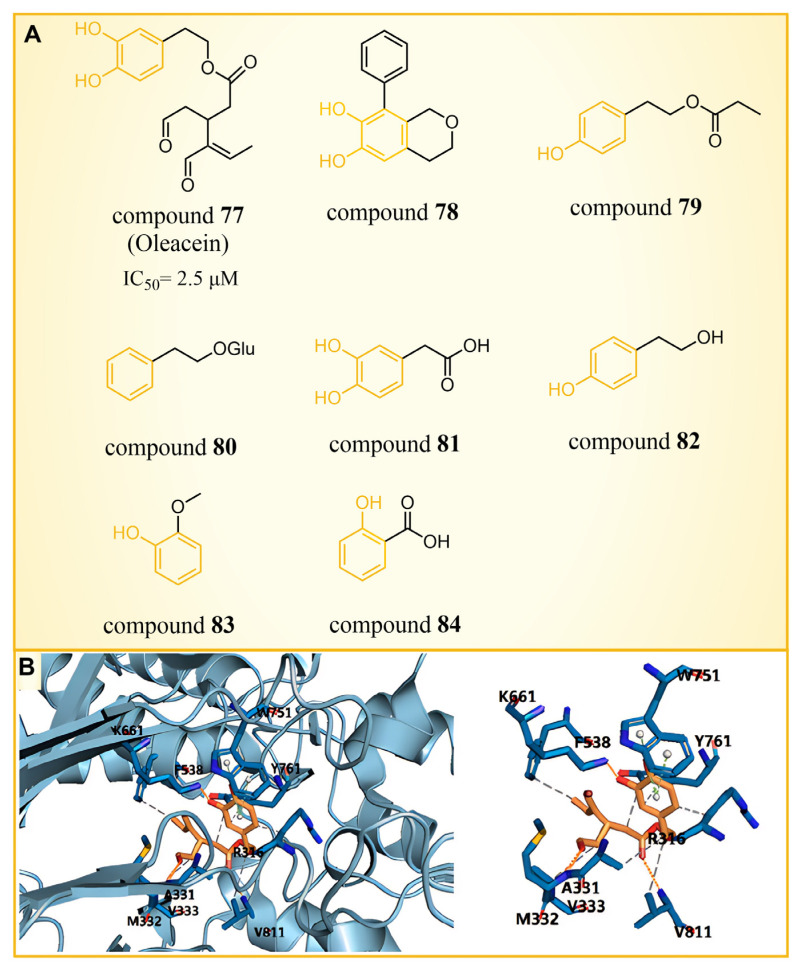
(**A**) The structures of phenolic compounds of compounds **77**–**84**; (**B**) complex structure of LSD1 upon binding to compound **77** (PDB: 2IW5, reprinted from Ref. [110]).

Olive oil, frequently referred to as a functional food [104], has demonstrated benefits in various diseases, such as cancer [105,106], diabetes [107], cardiovascular diseases [108] and neurodegenerative diseases [109]. A range of phenolic compounds derived from olive oil has demonstrated the capacity to inhibit LSD1. Cuà s et al. [110] confirmed that compound **77** (Figure 17A, ${\mathrm{IC}}_{50}^{\mathrm{LSD}1}$ = 2.5 μM, Oleacein), a biophenol secoiridoid naturally found in extra virgin olive oil, could inhibit LSD1 through a combined approach of molecular docking, molecular dynamics and binding free energy. Subsequent cell experiments verified that compound **77** suppressed the expression of transcription factor SOX2 (SEX determining Region Y-box 2) in cancer stem cells, and induced pluripotent stem cells (iPS) under the control of the remote enhancer targeted by LSD1. Based on the docking results (Figure 17A), they predicted that Oleacein would form hydrogen bond interactions with Lys661, Ala331 and Met332 of protein and hydrophobic contact with Arg316, Val333, Phe538 and Val811 in the binding pocket. In their docking predictions (Figure 17B), compound **77** was anticipated to engage in hydrogen bond interactions with Lys661. Pitsillou et al. utilized molecular docking methods to explore the binding properties of phenolic compounds with LSD1 and its variants, LSD2 and SETD7 [111]. Among 220 phenolic compounds, 208 successfully docked with LSD1. The top 25 compounds based on their docking scores (ranging from −72.7 to −97.3 kcal/mol) exhibited superior performance over the ORY-1001 control inhibitor. These compounds were able to establish interactions with residues Asp555 and Asp556 of LSD1. According to a Glide Energy data analysis, compounds **78**–**80** (Figure 17A, glucosides, flavonoids and secoiridoids) exhibited a stronger binding affinity compared to compounds **81**–**84** (Figure 17A, hydroxyphenylacetic acids, simple phenols, methoxyphenols and hydroxybenzoic acids). This study not only identifies promising phenolic lead compounds for LSD1, but also enriches the repertoire of natural product-based LSD1 inhibitors.

### 5.3. Resveratrol Derivatives

**Figure 18:**
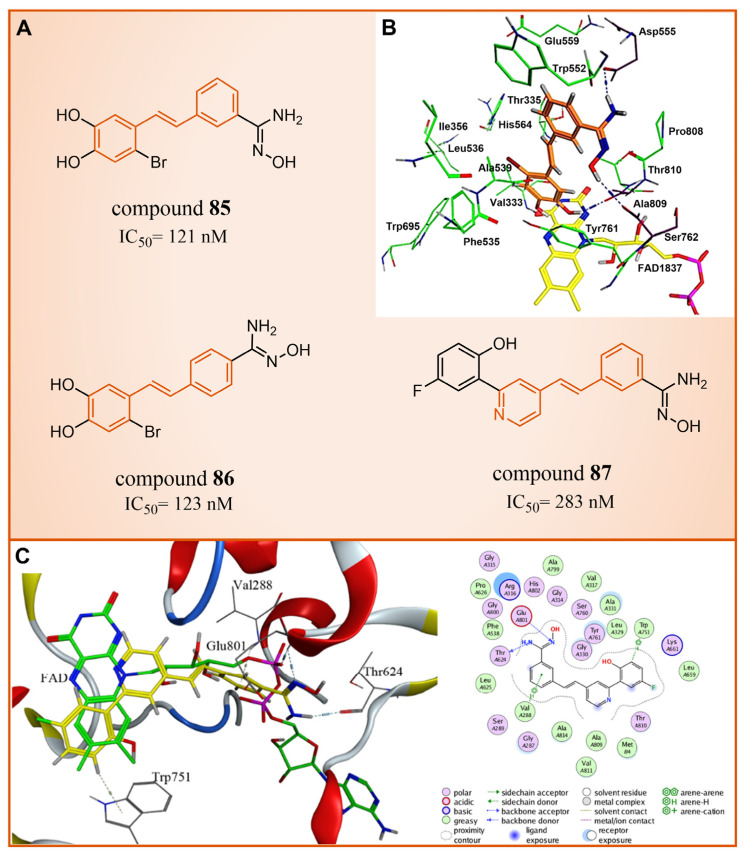
(**A**) The structures of resveratrol derivatives: compounds **85**–**87**; (**B**) complex structure of LSD1 upon binding to compound **85**; key amino acid residues and interactions are indicated (reprinted with permission from Ref. [113]. Copyright 2017 Elsevier); (**C**) Docking diagram of compound **87** with LSD1 (**left**) and 2D schematics of the protein–ligand interactions of compound **87** to LSD1 (**right**) (reprinted with permission from Ref. [114]. Copyright 2018 Elsevier).

In 2013, Abdulla et al. identified LSD1 as the target of resveratrol [112]. Subsequently, in 2017, our group designed and synthesized a series of reversible LSD1 inhibitors—resveratrol derivatives (Figure 18A) [113]. Among them, compound **85** (Figure 18A, ${\mathrm{IC}}_{50}^{\mathrm{LSD}1}$ = 121 nM, compound **4e**) and compound **86** (Figure 18A, ${\mathrm{IC}}_{50}^{\mathrm{LSD}1}$ = 123 nM, 4 m) exhibited superior inhibitory activities against LSD1 in enzyme assays. A high-content analysis showed that compounds **85** and **86** induced the dose-dependent increase in histone H3 dimethyl Lys4, but had no effect on the expression of LSD1 in MGC-803 cells. In addition, they can significantly increase the mRNA level of CD86 in MGC-803 cells, which is a substitute cell biomarker for LSD1 activity, which indicates that they may show LSD1 inhibitory activity in cells. According to the results of molecular docking (software: MOE 2015.10, Figure 18B), the amine of the amidoxime part of compound **85** formed a hydrogen bond with the carbonyl group of Asp555, and its hydroxyl group formed a hydrogen bond with the carbonyl group of Ser762. Moreover, this compound extensively formed hydrophobic interactions with Trp552, Ala539, Ala809, Thr810 and Pro808. These interactions enhanced the binding stability of the small molecules with protein. Building on this foundation, we synthesized a new series of new resveratrol derivatives in 2018, with compound **87** (Figure 18A, ${\mathrm{IC}}_{50}^{\mathrm{LSD}1}$ = 283 nM, 8c) emerging as the most potent LSD1 inhibitor [114]. Studies on enzyme kinetics and molecular docking (Figure 18C) indicated that compound **87** might be a competitive reversible inhibitor of the LSD1 cofactor FAD. Notably, the amidoxime segment of this compound formed a hydrogen bond with Thr624, and its phenyl group engaged in arene–H interactions with Val288, both contributing to the heightened binding stability of compound **87** with LSD1.

**Figure 19:**
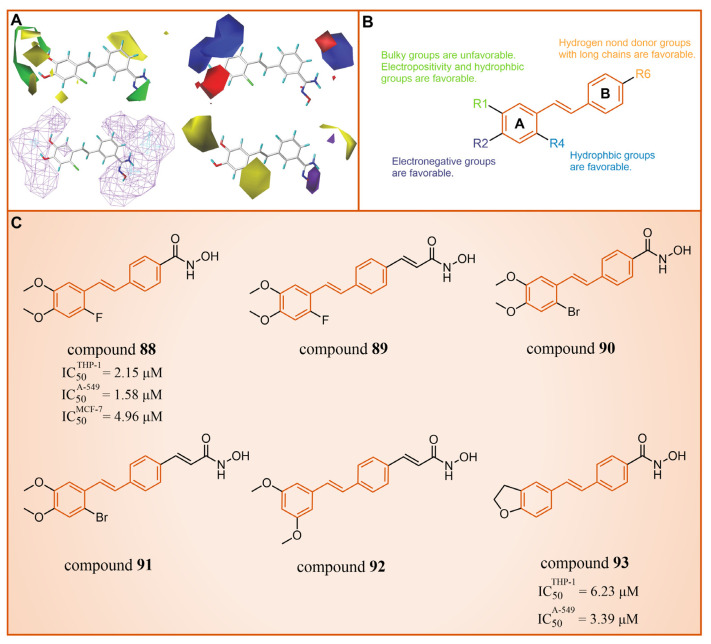
(**A**) 3D-QSAR contour maps visualize the effect of the introduced substituents on the biological activity (reprinted with permission from Ref. [102]); (**B**) structure–activity relationship (reprinted with permission from Ref. [102]); (**C**) the structures of resveratrol derivatives: compounds **88**–**93**.

Subsequently, our group constructed CoMFA [115,116] and CoMSIA [117,118] models with good statistical and predictive performance based on 34 resveratrol derivatives [113,114,119] with certain activity against LSD1 reported earlier (CoMFA: q^2^ = 0.682, r^2^ = 0.914, $R_{\mathrm{pred}}^{2}$ = 0.701; CoMSIA: q^2^ = 0.648, r^2^ = 0.949, $R_{\mathrm{pred}}^{2}$ = 0.824, where q^2^, r^2^ and $R_{\mathrm{pred}}^{2}$ represent the cross-validation coefficient, the non-cross-validation coefficient and the predictive correlation coefficient, respectively) [102]. The contour maps analysis (Figure 19A) and detailed structure–activity relationships (Figure 19B) guided modification suggestions for LSD1 resveratrol derivative inhibitors, leading to the design of six new derivatives, compounds **88**–**93** (Figure 19). Further ADME calculation results showcased high bioavailability and excellent drug-like properties for these new derivatives. Additionally, the 3D-QSAR model (software: Sybyl-X 2.0) predicted that compounds **88** and **90** had high pIC_50_ values in both the CoMFA and CoMSIA models (Figure 19A). It was fortunate that compounds **88** and **90** performed well in the anti-proliferation experiments in vitro (compound **88**: ${\mathrm{IC}}_{50}^{\mathrm{THP}\text{-}1}$ = 2.15 ± 0.33 μM, ${\mathrm{IC}}_{50}^{A\text{-}549}$ = 1.58 ± 0.14 μM, ${\mathrm{IC}}_{50}^{\mathrm{MCF}\text{-}7}$ = 4.96 ± 0.67 μM; compound **90**: ${\mathrm{IC}}_{50}^{\mathrm{THP}\text{-}1}$ = 3.39 ± 0.53 μM, ${\mathrm{IC}}_{50}^{A\text{-}549}$ = 6.23 ± 0.98 μM). The molecular docking results (software: MOE 2019.1002) demonstrated that these two inhibitors primarily interacted with residues Gly287, Val288, Ser289, Gly314, Arg316, Val317, Leu329, Gly330, Ala331, Leu659, Lys661, Trp751, Gly759, Ser760 and Tyr761. Notably, the R1 group was partially exposed to the solvent, suggesting caution in introducing a substituent with a larger volume, consistent with contour analysis results (Figure 19A).

### 5.4. Flavonoids

**Figure 20:**
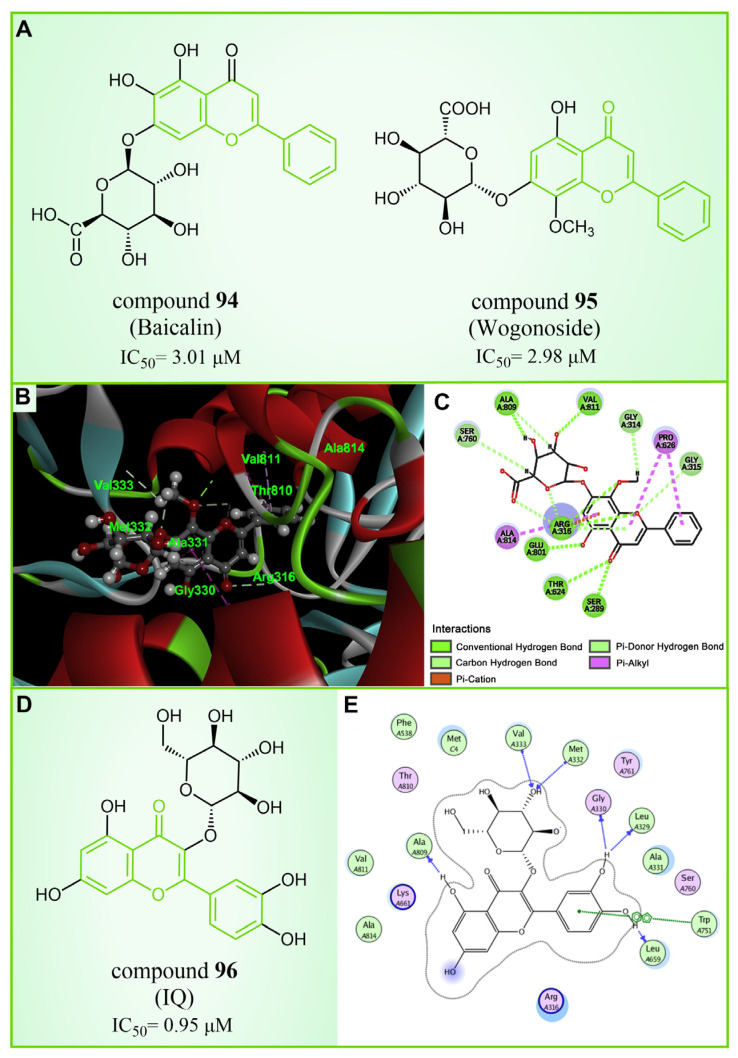
The structures and of flavonoids inhibitors. (**A**) Compounds **94** (Baicalin) and **95** (Wogonoside); (**B**) 3D docking model of compound **95** bound to LSD1(reprinted with permission from Ref. [123]. Copyright 2018 Elsevier); (**C**) 2D schematic of the docking model of compound **95** bound to LSD1(reprinted with permission from Ref. [123]. Copyright 2018 Elsevier); (**D**) compound **96** (IQ) and (**E**) 2D schematic of the docking model bound to LSD1(reprinted with permission from Ref. [124]. Copyright 2019 Elsevier).

Baicalin, an effective component of Coptidis Rhizoma, exhibits diverse pharmacological effects, including blood pressure reduction, tranquilization and antibacterial and anti-inflammatory properties [120,121]. In 2016, Zheng et al.’s research identified compound **94** (Figure 20A, ${\mathrm{IC}}_{50}^{\mathrm{LSD}1}$ = 3.01 μM, Baicalin) as the first LSD1 inhibitor derived from baicalin, demonstrating a certain inhibitory effect on LSD1 in cells [122]. Subsequently, in 2018, Han et al. [123] isolated six flavonoid LSD1 inhibitors from Scutellaria baicalensis Georgi via countercurrent chromatography (CCC), among which compound **95** (Figure 20A, ${\mathrm{IC}}_{50}^{\mathrm{LSD}1}$ = 2.98 μM, Wogonoside) showed superior inhibitory activity on LSD1. The molecular docking results revealed (software: MOE 2009, Figure 20B) that compound **95** could bind well to the FAD region of LSD1 and form numerous hydrogen bonds with Ser289, Arg316, Thr624, Glu801, Ala809 and Val811. Further experimental evaluation also demonstrated that compound **95** inhibited the migration and viability of MDA-MB-231 cells in a dose-dependent manner. In 2019, Xu et al. [124] selected 12 natural flavonoids LSD1 inhibitors for further research. The study investigated the inhibitory activity of 12 natural flavonoids on LSD1 and found that monosaccharide glycoside had greater inhibitory activity than aglycone lacking sugar. Among the flavonoids tested, compound **96** (Figure 20D, ${\mathrm{IC}}_{50}^{\mathrm{LSD}1}$ = 0.95 μM, isoquercitrin IQ), a monosaccharide glycoside, exhibited the strongest LSD1 inhibitory activity and induced apoptosis in MDA-MB-231 cells through LSD1 inhibition in a molecular docking study of flavonoid LSD1 inhibition and structure–activity relationship. The results of the molecular docking (Figure 20E) analysis indicated that the hydroxyl group located within the sugar part of compound **96** could form hydrogen bonds with Val333 and Met332. Additionally, the benzene ring of the flavone skeleton possessed the ability to form π–π stacking with Trp751, and the phenolic hydroxyl group of this part could form hydrogen bonds with Ala809, Gly330, Leu329 and Leu659. Flavonoid natural-product inhibitors merit further investigation based on their high inhibitory activity on LSD1.

### 5.5. Other Natural Products

**Figure 21:**
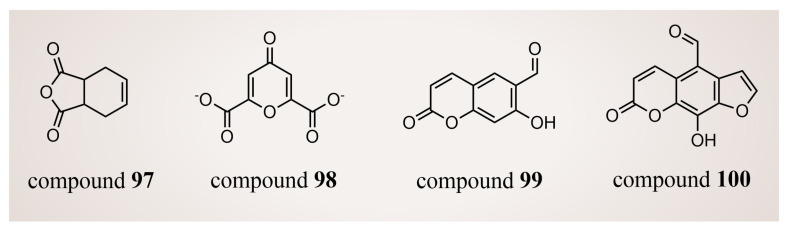
The structures of natural-product compounds **97**–**100**.

In 2018, Thai et al. [125] conducted a virtual screening of 2000 drug-like compounds from the TCM database (https://tcm.cmu.edu.tw, accessed on 15 February 2017) [126] with potential inhibitory effects on LSD1. Employing Lipinski’s rule, they selected the top 50 compounds based on favorable docking scores through molecular docking. Subsequent SMD simulations highlighted compounds **97**–**100** (Figure 21, compounds **4678324**, **10585521**, **14213968** and **6810**) as potential LSD1 inhibitors with nanomolar-level inhibition constants, determined by comparing the physical quantity, F_max_. In addition, according to the QSAR calculations, only compound **99** displayed slight toxicity, whereas the remaining three compounds were deemed non-toxic. Nevertheless, further in vitro and in vivo experiments are warranted to validate these findings.

Natural products present an extensive array of distinctive molecular scaffolds and serve as a valuable reservoir for the discovery of novel bioactive compounds. Among the myriad of natural-product LSD1 inhibitors, resveratrol derivatives, exemplified by compounds **85**–**87**, distinguish themselves. These derivatives demonstrate potent inhibitory activity against LSD1 at low micromolar levels, thereby positioning them as highly promising candidates for anticancer drug development.

## 6. Others

### 6.1. Thiourea Compounds

**Figure 22:**
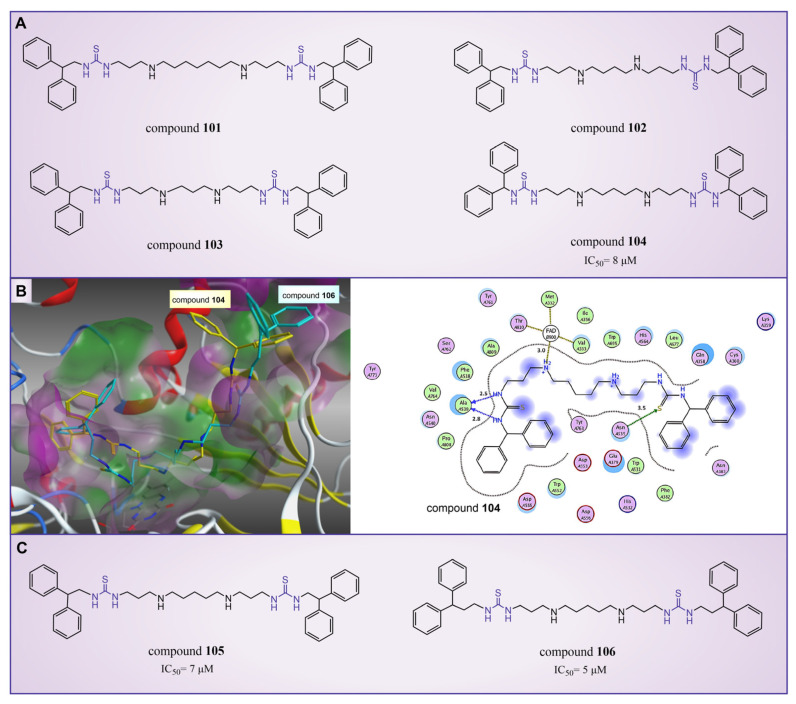
The structures of thiourea compounds. (**A**) Compounds **101**–**104**; (**B**) computer-predicted binding mode of compounds **104** and **106** in the LSD1 binding site (**left**), molecular interactions between LSD1 and compound **104** (**right**) (reprinted with permission from Ref. [128]. Copyright 2015 Elsevier); (**C**) compounds **105** and **106**.

In 2010, Sharma et al. reported a series of thiourea compounds that could effectively inhibit LSD1 [127]. In particular, compounds **101**–**103** (Figure 22A, compounds **25**–**27**) exhibited superior inhibitory activity against LSD1 in Calu-6 lung cancer cells [127]. Building on this foundation, several new thiourea derivatives were designed and synthesized by Shannon et al. in the same group in 2015 [128]. Among these, compound **104** (Figure 22A, ${\mathrm{IC}}_{50}^{\mathrm{LSD}1}$ = 8 μM, compound **6b**), compounds **105** and **106** (Figure 22C, ${\mathrm{IC}}_{50}^{\mathrm{LSD}1}$ = 7 μM and 5 μM, respectively, compounds **6c** and **6d**) displayed particularly potent inhibition against LSD1. Molecular docking results (software: GOLD 5.1, Figure 22B) suggested that compounds **104** and **106** shared similar binding modes with LSD1. Specifically, compound **104** could form hydrogen bonds with residues Asn535 and Ala539 of LSD1, and interact with Val333, Phe382, Phe538, Ala539, Trp552, Trp695, Tyr761, Val764 and Pro808 in the pocket of LSD1.

In addition, ongoing efforts within this group involve in vivo evaluation experiments for compounds **104** and **106**, as well as the synthesis and evaluation of other compounds within the thiourea series. This continued exploration aims to provide valuable insights into the therapeutic potential of thiourea compounds as LSD1 inhibitors.

### 6.2. Fenoldopam and Raloxifene

**Figure 23:**
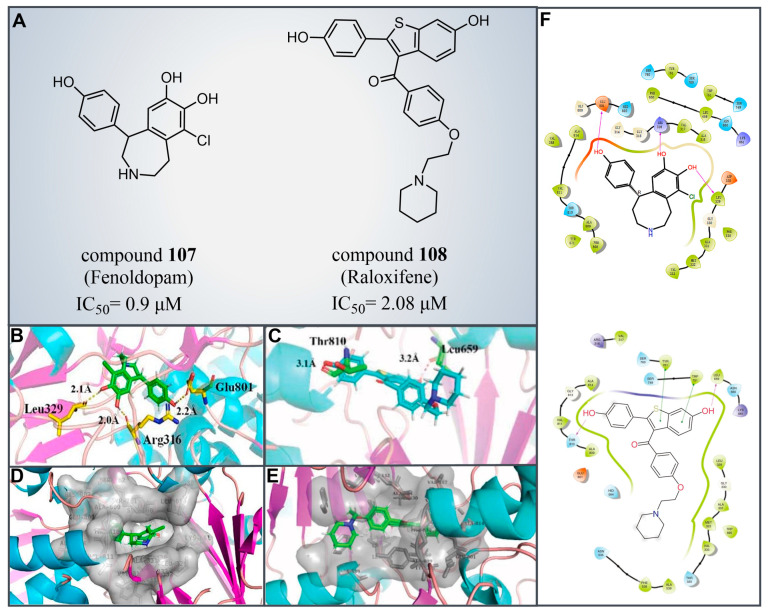
(**A**) The structures of compounds **107** (Fenoldopam) and **108** (Raloxifene); molecular docking results of (**B**) compounds **107** (reprinted with permission from Ref. [130]. Copyright 2021 Elsevier) and (**C**) **108** bonding to LSD1; hydrogen bonds and their distances are shown (reprinted from Ref. [131]). (**D**) Compounds **107** (reprinted with permission from Ref. [130]. Copyright 2021 Elsevier) and (**E**) **108** were buried in a hydrophobic pocket of LSD1 (reprinted from Ref. [131]); (**F**) 2D diagram depicting their interaction (**up**, compounds **107** reprinted with permission from Ref. [130]. Copyright 2021 Elsevier and **108**, **down**, reprinted from Ref. [131]) with LSD1.

Drug reutilization, with the advantages of a short development time and low development cost, is an attractive strategy for the treatment of human diseases [129]. In 2020, Zheng et al. screened LSD1 inhibitors from a complex library containing drugs approved by the US Food and Drug Administration (FDA) and identified the reversible inhibitor compound **107** (Figure 23A, ${\mathrm{IC}}_{50}^{\mathrm{LSD}1}$ = 0.9 μM, Fenoldopam), which can be used to treat renal cell carcinoma (RCC) [130]. Subsequent molecular docking results (software: MOE 2019.01, Figure 23B,D,F) showed that compound **107** would bind to the FAD region of LSD1 in a folded conformation. The three hydroxy groups on its benzene ring could form strong hydrogen bonding interactions with residues Arg316, Leu329 and Glu801 of LSD1, respectively. Meanwhile, the compound was ensconced within a hydrophobic pocket formed of residues Gly314, Gly315, Arg316, Val317, Ala318, Leu329, Gly330, Glu801 and Ala814. In the same year, Ma et al. from this group performed a high-throughput screening of a small compound library, leading to the identification of a novel LSD1 inhibitor, compound **108** (Figure 23A, ${\mathrm{IC}}_{50}^{\mathrm{LSD}1}$ = 2.08 μM, Raloxifene) [131]. This compound exhibited inhibitory effects on the proliferation and migration of RCC cells with LSD1 overexpressed. The molecular docking results (software: MOE 2015.10, Figure 23C,E,F) showed that compound **108** could bind to the hydrophobic pocket enclosed by residues Leu329, Gly330, Leu659, Ser749, Trp751, Tyr761, Ala809, Thr810, Val811, Gly813 and Ala814 of LSD1. It forms hydrogen bonds with Leu659 and Thr810, along with π–π stacking interactions with Trp751 and Tyr761. Given their status as already-marketed drugs, compounds **107** and **108** hold promise as scaffolds for developing novel LSD1 inhibitors for the treatment of renal cell carcinoma. 

### 6.3. (4-Cyanophenyl)glycine Derivatives

**Figure 24:**
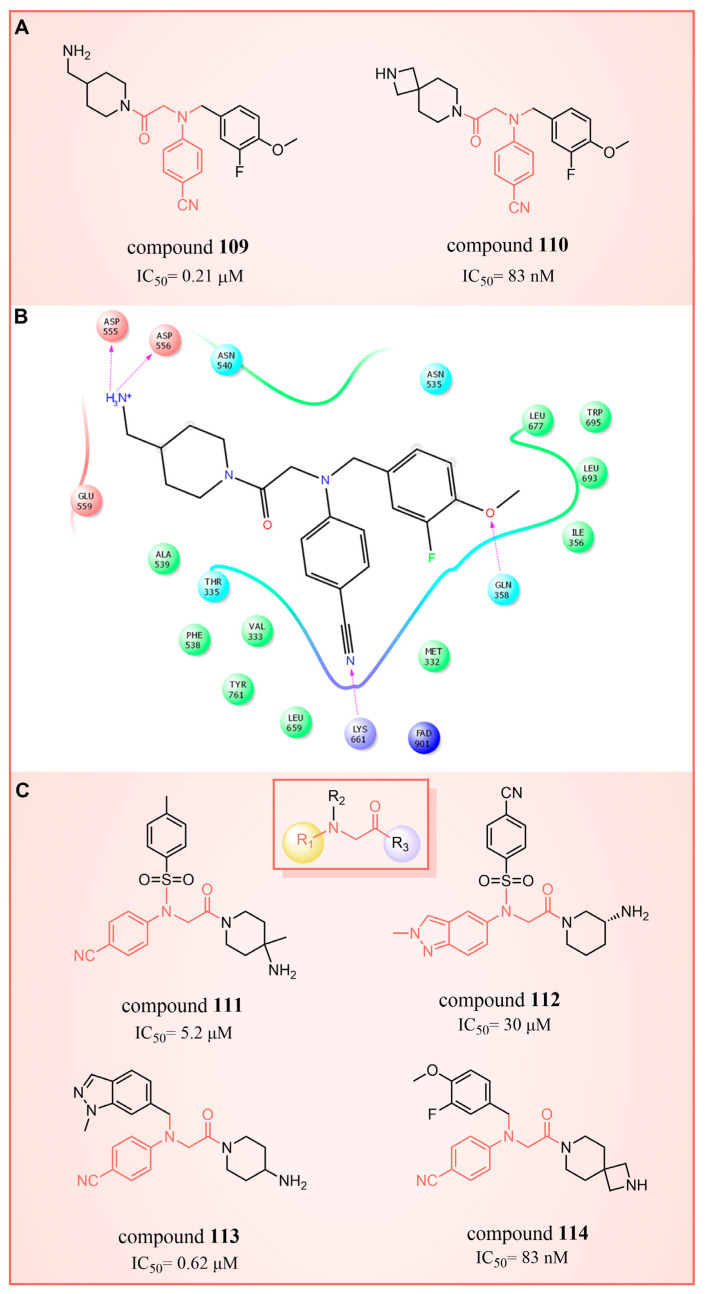
The structures of (4-cyanophenyl)glycine derivatives. (**A**) Compounds **109** and **110**; (**B**) 2D diagram depicting interaction of compound **109** with LSD1 (reprinted with permission from Ref. [132]. Copyright 2017 American Chemical Society); (**C**) compounds **111**–**114**.

**Figure 25:**
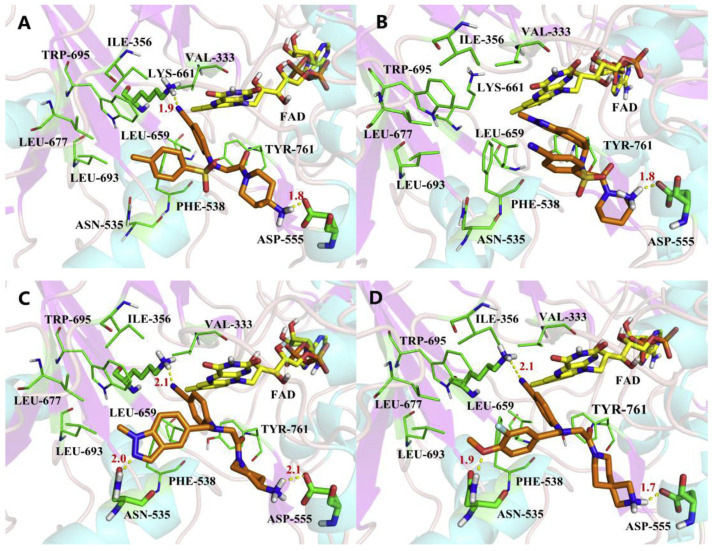
The proposed binding modes of LSD1 with compounds (**A**) **111**, (**B**) **112**, (**C**) **113** and (**D**) **114**, respectively (reprinted with permission from Ref. [133]. Copyright 2019 Elsevier).

In 2017, Mould et al. developed a series of (4-cyanophenyl)glycine derivatives, through high-throughput screening and a fragment library search, that exhibited inhibitory effects against LSD1 and had the potential to treat acute myeloid leukemia (AML) [132]. Among these derivatives, compounds **109** and **110** (Figure 24A, ${\mathrm{IC}}_{50}^{\mathrm{LSD}1}$ = 0.21 μM and 83 nM, respectively, compounds **30f** and **32**) demonstrated a more pronounced inhibition of LSD1. Later, they predicted the binding modes of compound **109** using molecular docking techniques (software: Glide 6.7, Figure 24B). The nitrile group of compound **109** formed a hydrogen bond with residue Lys661, and the ether oxygen moiety established a hydrogen bond with Gln358. The terminal alkaline N atom of the compound engaged in an ionic interaction with Asp555 and Asp556. Compound **110** was developed, which effectively inhibited LSD1 in biochemical, biophysical and cellular assays. However, these compounds strongly inhibited the human ether-a-go-go-related gene (hERG) cardiac ion channel, a deficiency that prevented further development of the series. To gain insight into the binding mechanism of (4-cyanophenyl)glycine derivatives with LSD1, Wang et al. conducted a comprehensive study in 2019, employing 3D-QSAR, molecular docking and molecular dynamics simulation techniques [133]. In their investigation of the binding mode between the four compounds **111**–**114** (Figure 24C, ${\mathrm{IC}}_{50}^{\mathrm{LSD}1}$ = 5.2 μM, 30 μM, 0.62 μM and 83 nM, respectively, compounds **10**, **15**, **21** and **29**) with the best molecular docking results and LSD1 (software: Gromacs 5.1.4, Figure 25), all compounds were found to bind to the substrate region of LSD1. The protonated nitrogen atoms at the end of the R1 groups in these four compounds could form hydrogen bonds with Asp555, and the substituents of their R2 groups could penetrate deeply into the hydrophobic pockets consisting of Ile356, Phe538, Leu677, Leu693 and Trp695. In addition, the R2 groups of compounds **113** and **114** also formed hydrogen bonds with Asn535, potentially contributing to the superior activity of these compounds compared to compounds **111** and **112**. The R3 groups of these four compounds were enveloped by FAD, Val333, Phe538, Leu659, Lys661, Trp695 and Tyr761, with the phenyl groups establishing hydrophobic interactions with these residues. At the same time, it can form π–π stacking interactions with FAD, Phe538, Trp695 and Tyr761. Subsequently, Wang et al. employed the CoMFA and CoMSIA methods to construct 3D-QSAR models with good statistical parameters. The conformational relationships explained by isopotential diagrams aligned well with the docking results [133]. The importance of Asp555 was demonstrated by the fact that the hydrogen bond between the above four compounds and Asp555 remained stable in the molecular dynamics simulations. Moreover, the cyano groups in these compounds formed a hydrogen bonding network with FAD and Lys661 through a conserved water molecule, significantly enhancing the stability of the protein–ligand complexes [134]. Binding free energy calculations emphasized the electrostatic nature of the main interaction between LSD1 and the ligand, with hydrophobic interactions playing a substantial role in the binding process. These results may help in the further design of reversible and effective LSD1 inhibitors for the treatment of AML.

### 6.4. Propargylamine Derivatives

**Figure 26:**
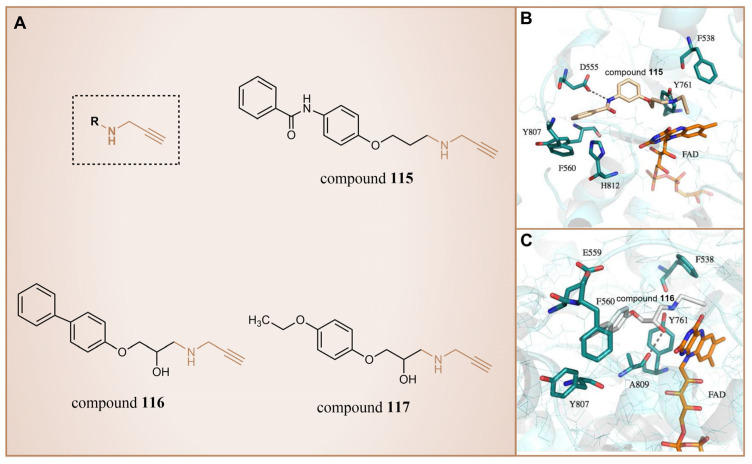
(**A**) The structures of propargylamine derivatives: compounds **115**–**117**; complex structure of LSD1 upon binding to (**B**) compounds **115** (reprinted with permission from Ref. [135]. Copyright 2013 American Chemical Society) and (**C**) **116** (reprinted with permission from Ref. [135]. Copyright 2013 American Chemical Society).

In 2013, Schmitt et al. designed and synthesized a series of propargylamine derivatives as covalent inhibitors of LSD1 using propargylamine as the warhead [135]. Meanwhile, the molecular docking study of compound **115** (Figure 26A, ${\mathrm{IC}}_{50}^{\mathrm{LSD}1}$ = 182.4 μM, compound **4a**) using MOE 2011.10 confined the warhead moiety (*N*-propargylamine group) to the N5 nitrogen atom of the tricyclic isoalloxazine ring of FAD according to the hypothesis of the covalent inhibition mechanism [136,137]. The results (Figure 26B) illustrated the formation of a hydrogen bond between the amine on the N-propargylamine warhead of this molecule and the residue Tyr761 on LSD1. Additionally, a hydrogen bond formed between the amide nitrogen atom of its benzamide moiety and the side chain of the residue Asp555. Furthermore, the aromatic substituent of compound **115** engaged in hydrophobic and T-shaped aromatic interactions with Phe558, Phe560, Tyr807 and His812. Following this, the researchers conducted a virtual screening of the Enamine database, which contains over 750,000 compounds, utilizing the *N*-propargylamine moiety as a substructure [135]. From the screening results, compounds **116** (Figure 26A, ${\mathrm{IC}}_{50}^{\mathrm{LSD}1}$ = 44 μM, T5342129(5a)) and **117** (Figure 26A) with similar structures (Figure 26C) were selected for in vitro testing, ultimately identifying compound **116** as an effective LSD1 inhibitor. Molecular docking studies (Figure 26C) revealed that the hydroxyl and amine groups on this molecule formed hydrogen bonds with residues Ala809 and Tyr761 of LSD1, respectively. Additionally, interactions occurred between the hydrophobic biaryl group and Phe560 and Tyr807 through T-shaped and edge-to-face alkyl–aryl interactions. This is the first demonstration that small-molecule inhibitors of LSD1 with a propargylamine structure can be realized. Although further optimization of selectivity for monoamine oxidase is required, such compounds may provide interesting mechanistic probes for further analysis of the functional effects of irreversible LSD1 inhibition in vivo.

### 6.5. Benzoylhydrazine Derivatives

**Figure 27:**
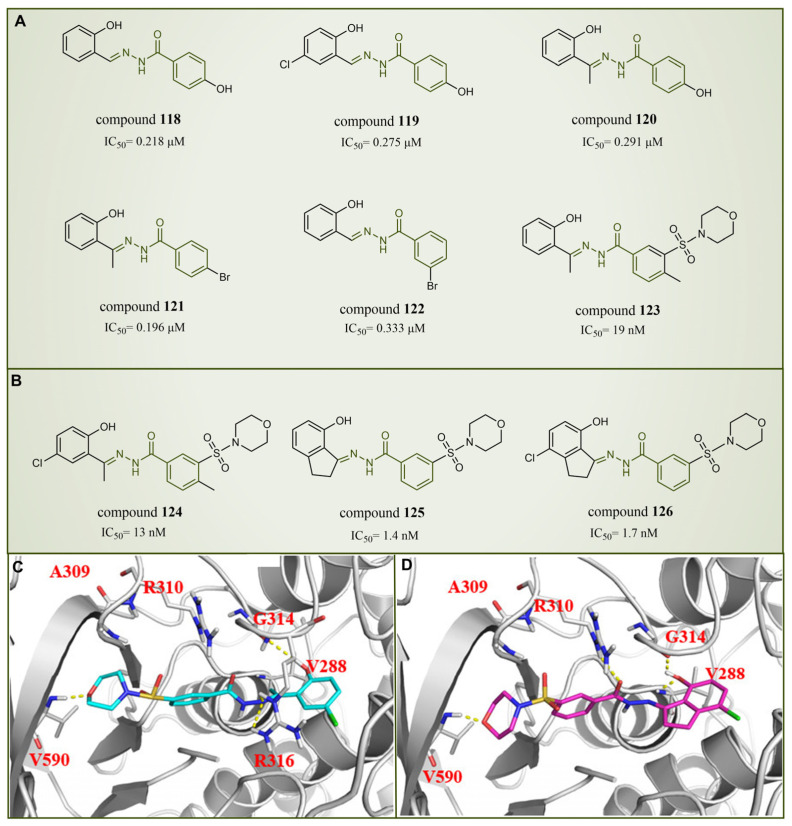
The structures of benzoylhydrazine derivatives. (**A**) Compounds **118**–**123**; (**B**) compounds **124**–**126**; (**C**) binding mode of compound **124** (reprinted with permission from Ref. [139]. Copyright 2016 Elsevier) and (**D**) compound **126** with LSD1; key residues are shown (reprinted with permission from Ref. [139]. Copyright 2016 Elsevier).

In 2013, Sorna et al. identified some novel *N*′-(1-phenylethylidene)-benzoylhydrazide derivatives with potential LSD1 inhibitory properties through the high-throughput virtual screening of a compound library containing 13 million small molecules, coupled with molecular docking [138]. Subsequent activity assays revealed that compounds **118**–**123** (Figure 27A, compounds **1**–**6**) exhibited notable inhibitory activity against LSD1. Building on this, 12 *N*′-(1-phenylethylidene)-benzoylhydrazide derivatives were designed and synthesized, with compound **124** (Figure 27B, ${\mathrm{IC}}_{50}^{\mathrm{LSD}1}$ = 13 nM, compound **12**) demonstrating promising biochemical activity. The binding mode diagram of compound **124** with LSD1 (Figure 27C) illustrated hydrogen bond formations with residues Gly314, Arg316 and Val590 of LSD1, thereby ensuring the binding stability of compound **124** and LSD1. In 2015, Zhou et al. optimized and synthesized a series of new (*E*)-*N*′-(2,3-dihydro-1*H*-inden-1-ylidene) benzoylhydrazide analogs based on compound **124**, evaluating their potential LSD1 inhibitory effects [139]. The evaluation results showed that compounds **125** and **126** (Figure 27B, ${\mathrm{IC}}_{50}^{\mathrm{LSD}1}$ = 1.4 nM and 1.7 nM, respectively, compounds **5a** and **5n**) exhibited significant LSD1 inhibitory activity. In addition, molecular docking studies (Figure 27C,D) further revealed a similar binding mode for compounds **124** and **126**. Both inhibitors established hydrogen bond interactions with residues Gly314 and Val590 of LSD1. Notably, the acyl group of compound **126** also established a hydrogen bond with Arg310, potentially contributing to its higher inhibitory activity. Compounds **125** and **126** stand out as promising inhibitors with substantial activity against LSD1. It is expected that more in-depth research will be conducted on the pharmacological properties and pharmaceutical properties of these compounds to explore their potential in clinical applications.

### 6.6. LSD1 Inhibitors Discovered through Artificial Intelligence Techniques

**Figure 28:**
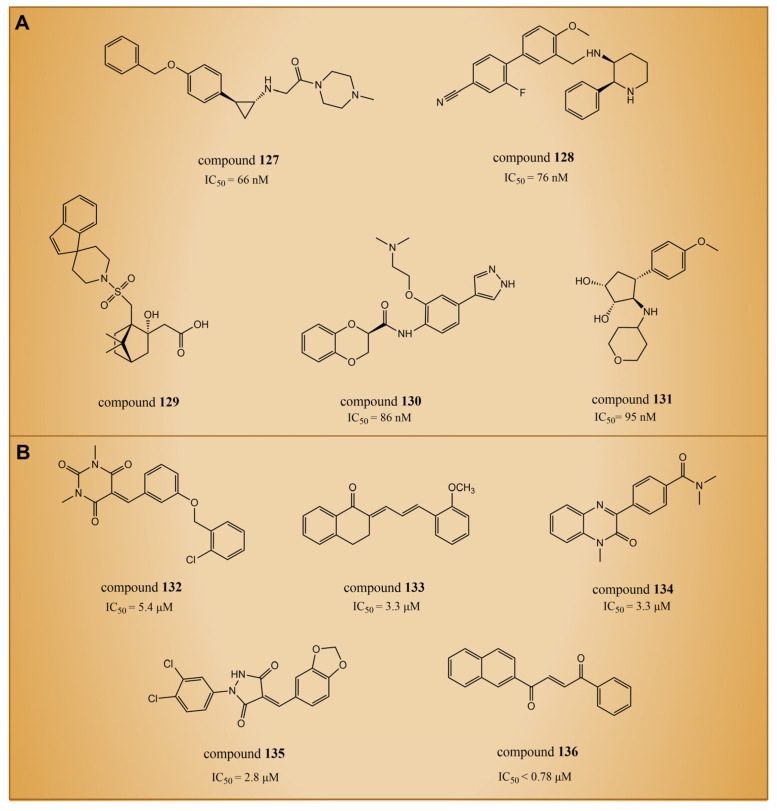
The structures of LSD1 inhibitors discovered through artificial intelligence techniques. (**A**) Compounds **127**–**131** (with the predicted IC_50_); (**B**) compounds **132**–**136**.

In 2021, Zhou et al. utilized a machine learning method to conduct virtual screening of LSD1 inhibitors [140]. The machine learning model was built based on a database of 931 small molecules with LSD1 inhibitory activity, and the activity predictions were made using Morgan molecular fingerprints. Then, the virtual screening of 300,000 molecules in the ZINC library identified compounds **127**–**131** (Figure 28A, the predicted ${\mathrm{IC}}_{50}^{\mathrm{LSD}1}$ = 66 nM, 76 nM, 86 nM and 1.7 nM, respectively, compounds **1**, **2**, **4** and **5**). This was the first instance of virtual screening for LSD1 inhibitors using machine learning and structural information alone. Due to the inherent uncertainty associated with model predictions in virtual screening, Wang et al. introduced a novel virtual screening method in 2022. This method is founded on the uncertainty of evidence in the Graphormer model for the identification of KDM1A/LSD1 inhibitors [141]. The Graphormer model underwent comprehensive evaluation, demonstrating advanced prediction performance, and thorough investigations into ranking performance and the calibration of evidence uncertainty. To showcase the practical integration of evidence uncertainty for more accurate compound selection, the evidence Graphormer model guided the retrospective screening of compounds using a time-division external dataset of KDM1A/LSD1 inhibitors. Subsequently, the trained model was applied to virtually screen an independent internal compound set. The top 50 compounds, selected through predictive sorting and UCB sorting, underwent experimental verification. Specifically, compounds **132**–**136** (Figure 28B, ${\mathrm{IC}}_{50}^{\mathrm{LSD}1}$ = 5.4 μM, 3.3 μM, 3.3 μM, 2.8 μM, <0.78 μM, respectively, compounds CPD25, CPD62, CPD82, CPD87 and CPD113) were chosen for subsequent experiments. In the experimental validation, these compounds were identified as hits with micromolar activity. Notably, compounds **132**, **133** and **136** exhibited moderate inhibitory activity against MAOs, and compounds **134**–**135** demonstrated acceptable selectivity. Further docking results indicated that the constructed Graphormer model exhibited logical consistency to a certain extent. The generated attention weight for each compound may offer valuable structure–activity explanations, providing insightful guidance for subsequent structural optimization. Therefore, the proposed uncertainty-guided virtual screening approach, based on the evidence-based Graphormer model, holds significant potential within a combined computational–experimental framework.

## 7. Conclusions and Prospects

Since the discovery of LSD1 in 2004, extensive research has been conducted on its inhibitors. As an effective tool for the rapid development of new drugs, CADD technology has also been applied to the search for LSD1 inhibitors. In this comprehensive review, we have systematically summarized the computationally relevant articles on LSD1 inhibitors since 2010.

The exploration of LSD1 inhibitors spans a diverse array of chemical scaffolds. The inhibitors, categorized by structural characteristics, include phenelzine derivatives, tranylcypromine (abbreviated as TCP or 2-PCPA) derivatives, nitrogen-containing heterocyclic (pyridine, pyrimidine, azole, thieno[3,2-b]pyrrole, indole, quinoline and benzoxazole) derivatives, natural products (sanguinarine, phenolic compounds and resveratrol derivatives, flavonoids, etc.) and others (thiourea compounds, Fenoldopam and Raloxifene, (4-cyanophenyl)glycine derivatives, propargylamine, benzohydrazide derivatives and inhibitors discovered through AI techniques). This demonstrates that CADD technologies play an important role in enriching the structural diversity of scaffolds for LSD1 inhibitors. The profound insights gained from these inhibitors lay a solid foundation for the development of innovative therapeutic strategies targeting LSD1 in various diseases, particularly cancer.

Covalent inhibitors, such as phenelzine and tranylcypromine, establish covalent bonds with the active site of LSD1, ensuring sustained inhibition. As noteworthy candidates, compounds **2** and **9** exhibit remarkable inhibitory activity against LSD1. However, certain safety concerns and off-target effects associated with TCP inhibitors pose challenges for clinical applications. In contrast, reversible inhibitors offer distinct safety advantages over irreversible ones. Consequently, the pursuit of novel reversible LSD1 inhibitors with enhanced activity and reduced toxicity has emerged as a prominent and challenging area of research.

Nitrogen-containing heterocycles, characterized by unique structures and rich biological activities, constitute a novel class of compounds extensively investigated as reversible LSD1 inhibitors. Rigorous screening and optimization efforts have identified several derivatives with significant inhibitory activity against LSD1, particularly noteworthy among them being compounds **21**, **50**–**52**. These compounds exhibit low nanomolar-level inhibitory activity, demonstrating a strong interaction with LSD1 and holding promise as highly effective inhibitors. Notably, compound **21** has shown good anti-tumor efficacy in small-cell lung cancer cell experiments, and has progressed to phase II clinical trials for the treatment of SCLC. The success of compound **21** underscores the potential of pyrimidine rings in drug design, urging further exploration of the structure–activity relationship of pyrimidine rings for developing additional pyrimidine compounds. However, compounds **61** and **63**, despite their excellent inhibitory activity against LSD1, face pharmacokinetic challenges that require structural optimization for enhanced clinical usability.

Natural products, offering unique molecular scaffolds, have proven to be a valuable resource for discovering new bioactive compounds. Resveratrol derivatives, exemplified by compounds **85**–**87**, stand out among natural-product LSD1 inhibitors, displaying potent inhibitory activity at low micromolar levels. The natural-product library itself encompasses a broader chemical space compared to synthetic small molecule libraries (such as covalent and nitrogen-containing heterocycle inhibitors) and often possesses high stereochemical complexity (Table 2). Leveraging natural products in drug development holds promise for providing crucial support in the exploration of LSD1 inhibitors.

In addition to the covalent, nitrogen-containing heterocyclic and natural LSD1 inhibitors, researchers have made exciting discoveries of other types of LSD1 inhibitors that exhibit potent inhibitory activity. Among them, compounds **125** and **126** exhibit significant anti-proliferative effects on LSD1 overexpressing cancer cells. In the development of LSD1 inhibitors, repurposing existing drugs, such as fenoldopam and raloxifene, has also yielded promising breakthroughs in LSD1 inhibitor development. This successful drug repurposing provides a compelling argument for the development of LSD1 inhibitors, highlighting the significance of drug repositioning strategies.

Furthermore, AI techniques have also made some progress in LSD1 inhibitor research, particularly in virtual screening, leading to the discovery of novel scaffolds with immense potential. Nevertheless, it is crucial to highlight that compound **110**, despite demonstrating strong inhibitory effects on LSD1, also exhibits a notable inhibition of the hERG myocardial ion channel. This aspect poses a substantial challenge to its continued research and development. Researchers are actively engaged in elucidating the mechanism of action of compound **110** and its interaction with hERG to either modify its structural attributes or devise innovative approaches aimed at mitigating or eliminating its inhibitory effect on hERG. This proactive approach is essential for propelling the advancement of the compound series and addressing the potential limitations associated with hERG inhibition.

In terms of the binding mode, the residues that interact with inhibitors bind mainly to the FAD region, including Val288, Ser289, Arg316, Val333, Thr335, Phe538, Thr624, Leu659, Lys661, Trp695, Trp751, Tyr761 and Ala809, where protein–drug intermolecular hydrogen bonds were easily formed at Val288, Arg316, Thr624 and Tyr761. The key residues interacting with the inhibitor-bound substrate region are mainly Thr335, Asn535, Phe538, Ala539, Asp555, Asp556, His564, Leu659, Lys661, Trp695 and Pro808, among which intermolecular hydrogen bonds are easily formed at Ala539 and Asp555. These modes of interaction should be in the focus of further LSD1 inhibitor studies.

The computational methods used in research in this field have changed significantly over the years. Early studies mainly relied on the pharmacophore model and the quantitative structure–activity relationship (QSAR) model. However, with the development of more CADD methods and the advent of artificial intelligence technologies, there has been a paradigm shift in the way in which researchers design and synthesize novel LSD1 inhibitors. These advanced techniques enable the more accurate prediction of pharmacokinetic profiles and drug–target interactions, leading to the discovery of novel stents that inhibit LSD1 activity. The application of CADD technology has led to the design and verification of effective LSD1 inhibitors, such as compounds **23**, **28** and **40**, showcasing the role of traditional CADD techniques like virtual screening, molecular docking, QSAR, molecular dynamics simulation, SMD and fragment-based drug design. The successful development of these inhibitors also relies on the application of CADD-related software. Among them, mainstream software tools for virtual screening and molecular docking include Glide, MOE, Sybyl and Discovery Studio. These software packages facilitate the simulation of molecular interactions, enabling the rapid screening of potential inhibitor molecules and the prediction of their binding modes with LSD1. Commonly used software for molecular dynamics simulation includes Amber, Gromacs and NAMD. These software packages are adept at simulating the dynamic behavior of proteins and inhibitors in a solution, providing nuanced details of interactions. Through molecular dynamics simulation, the binding mechanism of inhibitors with LSD1 and the dynamic process of inhibition can be further understood.

Concurrently, the exploration of LSD1 inhibitors confronts various challenges. For instance, non-specific irreversible inhibitors like phenelzine exhibit insufficient activity and limited selectivity, whereas TCP inhibitors manifest notable adverse reactions and substantial toxicity. Moreover, reversible inhibitors lack favorable pharmacokinetic characteristics. Particularly, a potential challenge lies in the development of resistance to LSD1 inhibitors, potentially constraining their long-term efficacy. In response to this challenge, researchers may need to explore alternative strategies, such as employing combination therapies or targeting multiple components of the epigenetic machinery. Additionally, a more in-depth investigation into the underlying mechanisms of LSD1 inhibition is imperative to facilitate the design of more selective and potent inhibitors.

Continued advancements in algorithms and computing power are paving the way for an increased role of machine learning and AI in drug research and development. These technologies, applied in drug design, screening, mechanism research and clinical trial assistance, provide efficient, accurate and reliable support for drug R&D. Notably, ISM001-055, a candidate drug discovered through InSilico Medicine’s AI drug development platform, has entered phase II clinical trials for idiopathic pulmonary fibrosis (https://classic.clinicaltrials.gov/ct2/show/NCT05975983, accessed on 30 December 2023). The Chemiverse Network module, employing various AI technologies and big data for drug design, has confirmed the effectiveness of a new CHK2 inhibitor in clinical trials for ovarian and breast cancer cells [142]. These achievements underscore the transformative potential of AI in drug research. The AI platform’s strengths in pharmacokinetic prediction, drug–target interaction and clinical trial design hold promise for addressing the challenges faced by LSD1 inhibitors. To propel LSD1 inhibitor research, innovative strategies must encompass improving selectivity, optimizing pharmacokinetic properties, employing combination therapies, investigating new mechanisms of action and leveraging AI in drug design.

In conclusion, the extensive of knowledge accumulated on LSD1 inhibitors, spanning synthetic and natural compounds, repurposed drugs and innovative drug discovery methodologies, is a testament to collaborative efforts across medicinal chemistry, biology and computational science. These endeavors hold exciting possibilities for the future of LSD1 inhibition in clinical settings.

## Figures and Tables

**Table 1:** Free energy result and energy components contributing to the binding free energy (kcal/mol).

	∆E_Vdw_	∆E_ele_	∆G_GB_	∆G_SA_	∆G (pred) *^a^*	K_i_ (exp) *^b^*
compound **2**	−43.88 (0.39) *^c^*	−22.18 (1.02)	22.41 (0.70)	−6.37 (0.017)	−50.04 (0.43)	0.059
compound **3**	−24.45 (0.27)	34.39 (0.99)	−33.74 (0.86)	−3.56 (0.0084)	−27.35 (0.32)	NA
compound **4**	−23.01 (0.33)	−20.39 (1.09)	12.89 (0.78)	−3.62 (0.0089)	−34.13 (0.48)	5.6

*^a^* The predictions do not include the contribution of conformational entropy; *^b^* μM; *^c^* standard error of the mean.

**Table 2:** Activity data of major compounds and their interactions with LSD1.

Type	Name	Molecular Structure	Key Residues	Interaction	Activity Data
Phenelzine derivatives	compound **2**	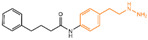	Thr624, Ser289, Val288, Val317, Val811, Ala814	hydrogen bond hydrophobic	K_i_ = 59 nM
TCP derivatives	compound **9**	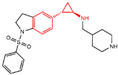	Asp555, Ala809, Tyr761, Phe538	hydrogen bond π–π stacking salt bridge	IC_50_ = 24.4 nM
Pyridine derivatives	compound **11**	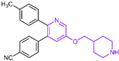	Asp555, Lys661	hydrogen bond	K_i_ = 29 nM
Pyrimidine derivatives	compound **20**	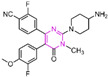	Asp555, Lys661	hydrogen bond salt bridge	IC_50_ = 0.3 nM
Triazole derivatives	compound **28**	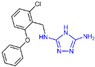	Ala539, Asn540, Asp555 FAD	hydrogen bond π–π stacking	IC_50_ = 15.1 μM
	compound **30**	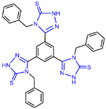	Arg316, Gly330, Ala331, Asn660, Lys661 Trp751	hydrogen bond π–π stacking arene–H	IC_50_ = 74 nM
Thiazole derivatives	compound **40**	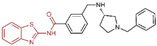	Asp555, Asp556, FAD	π–π stacking electrostatic interaction	IC_50_ = 18.4 μM
Pyrazole derivatives	compound **45**	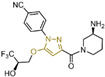	Gly287, Val288, Val333, Ser760	hydrogen bond	IC_50_ = 50 nM
Thieno[3,2-b]pyrrole derivatives	compound **54**	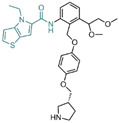	Asn535, Asp555, His564, Pro808	hydrogen bond	—
Indole derivatives	compound **57**	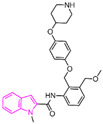	Asp555, FAD	hydrogen bond π–π stacking	IC_50_ = 4 μM
	compound **61**	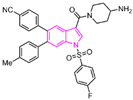	Met332, Val333, Ile356, Ala539, Trp552, Asp555, His564, Lys661, Trp695 Ala809, FAD	hydrogen bond π–π stacking hydrophobic	IC_50_ = 50 nM
Quinoline derivatives	compound **63**	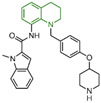	Asp555, Asp556, Glu559, FAD	hydrogen bond π–π stacking	IC_50_ = 50 nM
	compound **65**	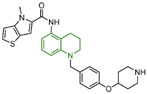	Cys360, Asp375, Trp695, FAD	hydrogen bond π–π stacking hydrophobic	IC_50_ = 0.19 μM
Phenyl oxazole derivatives	compound **75**	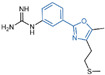	Arg316, Glu308, Arg310	hydrogen bond π–cation interaction	IC_50_ = 9.5 μM
Sanguinarine	compound **76**	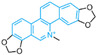	Lys661, Tyr761, Leu659, Lys661, Thr335, Ala809, Val811	hydrogen bond hydrophobic	IC_50_ = 0.4 μM
Phenolic compounds	compound **77**	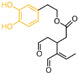	Lys661, Ala331, Met332, Arg316, Val333, Phe538, Val811	hydrogen bond hydrophobic	IC_50_ = 2.5 μM
Resveratrol derivatives	compound **85**	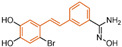	Asp555, Ser762, Ala809, Trp552, Ala539, Ala809, Thr810, Pro808	hydrogen bond hydrophobic	IC_50_ = 121 nM
Flavonoids	compound **96**	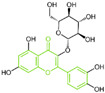	Trp751, Ala809, Val333, Met332, Gly330, Leu329, Leu659	hydrogen bond π–π stacking	IC_50_ = 0.95 μM
Thiourea compounds	compound **104**	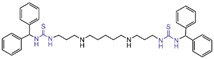	Asn535, Ala539, Val333, Phe382, Phe538, Ala539, Trp552, Trp695, Tyr761, Val764, Pro808	hydrogen bond hydrophobic	IC_50_ = 8 μM
Fenoldopam and Raloxifene	compound **107**	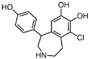	Arg316, Ala814, Leu329, Glu801, Gly314, Gly315, Arg316, Val317, Ala318, Leu329, Gly330, Glu801	hydrogen bond hydrophobic	IC_50_ = 0.9 μM
	compound **108**	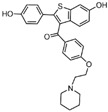	Leu329, Gly330, Leu659, Ser749, Trp751, Tyr761, Ala809, Thr810, Val811, Gly813, Ala814, Leu659, Thr810, Trp751 Tyr761	hydrogen bond π–π stacking hydrophobic	IC_50_ = 2.08 μM
(4-Cyano phenyl) glycine derivatives	compound **114**	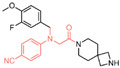	Asp555, Ile356, Phe538, Leu677, Leu693, Trp695, Asn535, FAD, Val333, Leu659, Lys661, Tyr761	hydrogen bond π–π stacking hydrophobic	IC_50_ = 83 nM
Propargylamine Derivatives	compound **116**	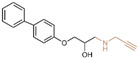	Ala809, Tyr761, Phe560, Tyr807	hydrogen bond T-shaped and edge-to-face alkyl–aryl interactions	—
Benzoyl Hydrazine Derivatives	compound **126**	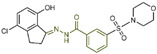	Gly314, Val590, Arg310	hydrogen bond	IC_50_ = 1.7 nM
